# Identification of genomic regions and candidate genes associated with soybean seed sugars in a RIL population

**DOI:** 10.3389/fpls.2026.1785097

**Published:** 2026-06-18

**Authors:** Nacer Bellaloui, James R. Smith, Jeffery D. Ray, Neeraj Kumar, Abdelraheem Abdelraheem, Chunda Feng, Alemu Mengistu

**Affiliations:** 1 USDA, Agriculture Research Service, Crop Genetics Research Unit, Stoneville, MS, United States; 2 USDA, Agriculture Research Service, Crop Genetics Research Unit, Jackson, TN, United States

**Keywords:** Functional genomics, soybean seed, seed sugars, sugars genes, sugars pathways, sugars QTL

## Abstract

Soybean seed sugar profiling determines the quality of soymeal for humans and livestock nutrition. Seed sucrose is desirable for taste and flavor, whereas raffinose and stachyose are indigestible, and therefore undesirable for humans and monogastric animals. Thus, identifying the genomic regions and genes controlling sugar type and level in the seed is desirable for developing optimal levels of each sugar. The objective of this research was to locate the genomic regions for seed sugars content and further identify potential candidate genes involved in sugar metabolism. A segregating mapping population (recombinant inbred lines, RILs) for heat tolerance was developed from a cross between DS25-1 (heat-tolerant parent) and DT97-4290. Both DS25–1 and DT97–4290 are maturity group IV. 201 RILs, parents, and checks were planted in field experiments conducted in 2018 and 2019 without irrigation at Stoneville, MS. Composite interval mapping (CIM) was performed to identify QTLs using a high-density linkage map with 8,445 polymorphic SNP markers. A logarithm of odds (LOD) value of ≥ 2.5 was used as the QTL significance level. Results showed that a total of 19 QTL for seed sugars, among which 12 were novel, were identified. A total of 8 QTL was identified for sugars in 2018. Four QTL (*qSu-Gm03-01*-2018, *qSu-Gm05-02*-2018, *qSu-Gm11-03*-2018, and *qSu-Gm19-04*-2018) were identified for sucrose on chromosomes (chrs) 3, 5, 11, and 19, respectively. Two QTL (*qRaf-*Gm06-*01–*2018 and *qRaf-Gm20-02*-2018) were detected for raffinose on chrs 6 and 20, respectively, and two QTL were identified for stachyose (*qSta-Gm06-01–*2018 and *qSta-Gm19-02*-2018) on chrs 6 and 19, respectively. In 2019, four QTL on chrs 5, 9, 17, 19; four QTL on chrs 6, 14, 19, and 20; and three QTL on chrs 3, 13, 19, were identified for sucrose, raffinose and stachyose, respectively. More than 50 candidate genes within each QTL interval were identified that are involved in sugar metabolic pathways. The SNP markers, QTLs, and candidate genes identified in this study provide new information that was previously unknown. The molecular markers will potentially assist breeders to select higher sucrose and lower stachyose that will increase soymeal nutritional qualities, enhancing farmer economic profit and minimizing production cost.

## Introduction

Soybean is a major crop in the USA and worldwide, and its seed is a source of protein, oil, and sugars. Seed protein content ranges from about 36% to 40%, oil from 18% to 23%, and sugars are about 24% (Krober and Cartter, 1962; Bellaloui et al., 2009, Bellaloui et al., 2010). About 47% of the carbohydrates are soluble, including glucose, fructose, sucrose, and raffinose family oligosaccharides (RFOs) (Hymowitz and Collins, 1974; Liu, 1997). Among these soluble sugars, sucrose, which is the most abundant sugar, accounts for 2.5 to 8.2%, followed by stachyose (1.4–4.1%), and then raffinose (0.1-0.9%) (Zeng et al., 2014). Both glucose and fructose are present in trace amounts (<1%) (Hymowitz and Collins, 1974; Liu, 1997). Sucrose is a desirable sugar because of its contribution to taste and texture of soy foods such as tofu, soymilk, and other soy foods (Kumar et al., 2011). Seed sucrose content is inversely correlated with firmness of tofu (Poysa and Woodrow, 2002) and the ratio of sucrose to stachyose is important for the natto fermentation process (Taira et al., 1990). Both raffinose and stachyose are undesirable sugars for consumption (Hata et al., 1991) as they cause flatulence and diarrhea in non-ruminant animals such as pigs and chickens, because these animals lack the *α (1, 6)-galactosidase* enzymes (Gitzelmann and Auricchio, 1965; Zeng et al., 2021). Therefore, identifying genomic regions controlling sugars in seeds is essential to select for higher sucrose content and for lower levels of raffinose and stachyose. On the other hand, RFOs are involved in seed desiccation and stress tolerance (Blackman et al., 1992; Bellaloui et al., 2010; Redekar et al., 2020), tolerance to freezing and seed longevity (Horbowicz and Obendorf, 1994; ElSayed et al., 2014). RFOs are formed from sucrose through a series of additions of galactinol. Galactinol synthase converts galactinol and myo-inositol, the main precursors, to form RFOs. Galactinol synthase converts myo-inositol and uridine diphosphate galactose into galactinol, and raffinose synthase converts sucrose and galactinol into raffinose (Keller and Pharr, 1996; ElSayed et al., 2014; Knizia et al., 2023). Also, RFOs are involved in several metabolic and signal systems such as signal transduction pathways (Xue et al., 2007), export of specific mRNAs (Okada and Ye, 2009), and transport across membranes (Thole and Nielsen, 2008). Sugars are affected by environment and growing conditions, including drought, heat, and soil conditions (Blackman et al., 1992; González et al., 1995; Wijewardana et al., 2019). For example, stachyose was shown to be increased by drying seeds (Blackman et al., 1992) and sucrose synthase activity was shown to decline during drought, resulting in a significant increase in sucrose (González et al., 1995).

In spite of the role of sugars in physiological and biochemical pathways, which are well documented (Marschner, 1912; ElSayed et al., 2014; Brown et al., 2021; Knizia et al., 2023), useable molecular markers, the genomic regions and candidate genes involved in controlling the level and profile of seed sucrose and RFOs, have not been completely well characterized or identified. Previous research reported more than 53 QTLs associated with seed sugars using QTL mapping from biparental populations and natural populations through genome-wide association studies (GWAS) (Brown et al., 2021; Kassem, 2021). On the other hand, only a few studies identified candidate genes within these QTL regions (Zeng et al., 2014; Kassem, 2021). For example, *Glyma.01g127600* encodes for a protein phosphatase on chr. 1, *Glyma.03g019300* encodes for a MADS-box protein, *Glyma.03g064700* encodes for a phosphatidylinositol monophosphate-5-kinase on chr. 3, and *Glyma.06g194200* encodes for a gibberellin-regulated protein on chr. 6 (Kassem, 2021; Salari et al., 2021). Knizia et al. (2023), using a ‘Forrest’ by ‘Williams 82’ recombinant inbred line soybean population with 309 lines, were able to identify a total of 26 QTL controlling seed sugar content on 16 soybean chromosomes. They were also able to identify 57 candidate genes, among which was a cluster of four genes involved in the sugar pathway on chr. 17.

Xu et al. (2022) reported 57 QTLs that were identified using databases and published papers. They found that transcriptome sequencing at the early, middle, and late stages of seed development revealed 158, 109, and 329 differentially expressed genes, respectively. Wen-Jin et al. (2022) were able to identify three candidate genes (*Glyma.19G146800*, *Glyma.19G122500*, and *Glyma.19G128500*) in the genetic fragments introduced from a wild soybean. Sequence comparisons between the two CSSL (chromosome segment substitution lines) parents SN14 and ZYD00006 revealed a SNP mutation in the coding sequence of the gene *Glyma.19G122500*, leading to an amino acid sequence mutation affecting protein structure. Hu et al. (2023), using GWAS and a population of 323 soybean accessions, identified 31,245 single-nucleotide polymorphisms (SNPs) with minor allele frequencies and were able to identify 72 QTLs associated with individual sugars and 14 identified with total sugars. They identified 10 candidate genes, among which were eight genes involved in sugar metabolism in soybean, with similar functions in *Arabidopsis*, and two other genes associated with sugar composition.

Maughan et al. (2000), using 149 F_2_ individuals, mapped 10 QTLs associated with seed sucrose content on 7 chromosomes (5, 7, 8, 13, 15, 19, and 20). These QTLs explained 6.1 to 12.4% of the total phenotypic variation. In a segregating population (115_F2:10_ lines; and 117_F2:10_ lines)using SSR markers, Kim et al. (2005, Kim et al., 2006) identified 6 QTLs for raffinose, stachyose, and sucrose on chromosomes 2, 11, 12, 16, and 19. Skoneczka et al. (2009), using two F_2_ populations (PI870139 × PI200508 and PI2435459 × PI200508) were able to identify a major QTL on chromosome 6 for low-stachyose and high-sucrose contents. They reported that this QTL explained 88–94% of the phenotypic variation for stachyose and 76% of the variation for sucrose content. Further, Zeng et al. (2014) mapped three sucrose QTLs located on chromosomes 5, 9, and 16 that explained 46, 10, and 8% of the phenotypic variation, respectively. Wang et al. (2014) used a population of 170 F_2:3_ lines and identified 11 QTLs for sugars, including one QTL for glucose, 3 QTLs for fructose and sucrose, and 2 QTLs for raffinose and stachyose. Akond et al. (2015) used 92 F_5:7_ RILs derived from MD965722 × ‘Spencer’ to identify 14 QTLs on 8 chromosomes (1, 3, 6, 9, 12, 14, 15, and 16), including 3 for sucrose, 7 for raffinose, and 4 for stachyose. Salari et al. (2021) identified four QTLs associated with sucrose and raffinose in a mapping population of 140 RILs derived from ‘IA3023’ × LD02-5585.

Liu et al. (2023) used a RIL population derived from a cross between ZD27 and HF25, which have different levels of sucrose and soluble sugars. They identified 16 QTLs related to sucrose and soluble sugar, among which was a major QTL associated with sucrose, *qSU1901*, that explained 10.6%– 13.2% of the phenotypic variation. They also conducted a transcriptomic analysis and studied the expression of key genes involved in sucrose and soluble sugar transport and metabolism. By combining QTL-Seq and RNA-Seq data, they reported a gene regulatory network involving 233 candidate genes. Maughan et al. (2000) used an F_2_ population with 178 polymorphic markers and identified 17 sucrose-specific marker loci that were mapped to 7 different genomic regions. Zeng et al. (2014) identified 8 sucrose QTLs on five chromosomes using SSR and SNP markers. Wang et al. (2014) identified 11 QTLs associated with five soluble sugar components using an F_2_ population derived from soybean cultivar V97–3000 and V99-5089. Although these early findings provided a foundation for subsequent gene localization, the localization interval and accuracy were limited due to the genomic technological restrictions of earlier research methods, and the lack of cultivar availability with optimum low raffinose and stachyose, and higher sucrose.

Based on the above literature, it is clear that higher sucrose and reduced raffinose and stachyose is desirable and obtainable, but the goal of identifying useable molecular makers that can be utilized in breeding to produce soybean germplasm/cultivars with optimum higher sucrose and optimum reduced raffinose and stachyose has not yet been achieved. Jamison, (2024); Hu et al. (2023) reported that although extensive research had been conducted on QTL associated with seed protein and oil, relatively limited research had been done on identifying molecular markers utilized in breeding, QTL, and candidate genes associated with seed sugars. Therefore, the objective of this research is to identify genomic regions controlling sucrose, raffinose, and stachyose contents in soybean seed, and identify candidate genes involved in metabolic pathways of sugars. This study used a 201 RIL (recombinant inbred line) population derived from a cross between DS25–1 and DT97-4290, which are segregating for heat tolerance, and used a whole-genome QTL mapping approach followed by composite interval mapping (CIM) to identify new markers associate with sucrose, raffinose, and stachyose.

## Materials and methods

### Plant materials and experimental design

The RIL population was developed using a cross between DS25-1 (Chebrolu et al., 2016; Narayanan et al., 2020) and DT97-4290 (Paris et al., 2006). Both DS25–1 and DT97–4290 are maturity group IV. Based on maturity, a subset of 201 recombinant inbred lines (RILs) was chosen for field experiments conducted in 2018 and 2019. Parental lines along with 12 checks were also included each year. This RIL population was designed specifically to grow under a high heat environment such as of the midsouth USA, and fulfil our goals of finding molecular markers and candidate genes that are related to seed sugars under specifically high heat and water deficient environment. The experiments were conducted in a RCBD with two replications at Stoneville, MS, under non-irrigated (rainfed) growing conditions. Each plot was a single row 3 m long with a row spacing of 0.9 m. Plots were bordered on each side by adjacent single-row plots or border fill rows. Hence, all plots were bordered on both sides. The planting dates were 20 April 2018 and 22 April 2019. A seeding rate of 25 seed/m/row was used. A combination of Reflex (Syngenta, DE, USA) and Select (Valent, Corporation, CA, USA) herbicides, with machine cultivations and hand hoeing, were used for weed control. Each plot was bulk-harvest by hand within two to four days after R8 (full-maturity). Stand counts prior to harvest were not taken, but all plots were visually rated as having a full stand. We targeted R8 growth stage as reported by Fehr and Caviness (1977) in both years. Harvesting the seeds shortly after R8 (harvest maturity) would coincide with what the growers normally do and ship at the grain elevator. Harvested plant bundles from each plot were stored in an air-conditioned room until threshed in a machine bundle thresher shortly thereafter. Threshed seed was stored at 21°C and 60% relative humidity until all plots were harvested and threshed, after which each seed lot was sampled to estimate levels of seed sugars (sucrose, raffinose, and stachyose).

### Sugar phenotyping

Soybean seed of RILs, parents, and checks were harvested and analyzed for seed sugar content. Briefly, about 25 g from each seed sample were ground by a Laboratory Mill 3600 (Perten, Springfield, IL), and sucrose, raffinose, and stachyose were analyzed by near infrared reflectance (Wilcox and Shibles, 2001; Bellaloui et al., 2010) using a diode array feed analyzer AD 7200 (Perten, Springfield, IL). Calibrations were developed by the University of Minnesota, using Perten’s Thermo Galactic Grams PLS IQ software, and established according to Biermann and McGinnis (1989) and Churms et al. (1982). This method was based on the conversion of sucrose to fructose and glucose using the enzyme Invertase (β-fructosidase), and then the glucose produced was measured by the hexokinase method (Biermann and McGinnis, 1989). Sucrose measurement was based on a dry matter basis (Wilcox and Shibles, 2001; Boydak et al., 2002; Bellaloui et al., 2010).

### Genotyping-by-sequencing data processing and map construction

Leaf tissue from 201 RILs and two parental lines (DS25–1 and DT97-4290) was collected and lyophilized. DNA was extracted using a Maxwell 16™ automated system (Promega) and genotyped using the GBS method (Elshire et al., 2011) by LGC Genomics GmbH (https://www.biosearchtech.com/) with MsII and Illumina NextSeq (2 × 150 bp) sequencing. Raw sequences were aligned to the *Glycine max* “Williams 82” reference genome (https://www.soybase.org/). Reads were processed, barcodes and adapters removed, and SNP calling was done using Freebayes v1.92-16. Monomorphic, heterozygous, and low-quality SNPs (>15% missing data) were excluded. Missing data for SNPs with <15% were imputed using the LD-kNNi method, which is based on a k-nearest-neighbor genotype (Money et al., 2015) in TASSEL software. (TASSEL, n.d., https://www.maizegenetics.net/tassel). Detailed methods for cleaning SNP data have been provided in our previous study (Kumar et al., 2026). For constructing the linkage map, the genotyping data of all the 247 RILs were converted into the ABH allele format, where “A” represents the allele from DS25-1, “B” represents the allele from DT97-4290, and “H” denotes heterozygous loci. Altogether, 8,890 polymorphic SNP markers were used for map construction using R/qtl (Broman et al., 2003). None of the SNPs exhibited segregation distortion (SD) at Chi-square *P* ≤ 0.05, only ~ 0.50% SNPs showed SD at Chi-square *P* ≤ 0.01, which were removed. Approximately ~396 SNPs were discarded because of redundancy (duplicate SNPs), markers with small linkage groups (≤ 5 markers), and unliked LGs. Finally, 22 LGs were formed with 8,445 SNPs using LOD score ≥ 3.0 with a maximum recombination frequency of 0.35, corresponding to 20 soybean chromosomes. Genetic distance between the markers was calculated in centiMorgans (cM) using the Kosambi mapping function (Kosambi, 1943). A total of 8,445 polymorphic SNPs were mapped with R/qtl (Broman et al., 2003; Broman et al., 2019), resulting in 22 linkage groups corresponding to the soybean chromosomes. A high-density genetic map was constructed with a total length of 2,519.91 cM and average marker distance was 0.43 cM (**Supplementary Figure 1**). Although highest marker density was observed on Gm04 (1 marker/0.09 cM), while the lowest density was on Gm12 (1 marker/0.97 cM). In addition to that, the highest number of markers were mapped at Gm18 (999 SNPs), while lowest mapped on Gm12 (137 SNPs). Detailed methods for map construction were provided in our previous study (Kumar et al., 2026).

### Mapping of QTLs for sucrose-related traits

QTL analysis was performed on the DS25–1 x DT97–4290 RIL population utilizing genomic data of 8,445 markers and phenotypic data collected in 2018 and 2019 on seed sucrose, raffinose, and stachyose. The mean values of each individual environment of each phenotypic trait were used for performing QTL analysis. For QTL detection, a composite interval mapping (CIM) module was used following the *R/qtl* program (Broman and Sen, 2009; Broman et al., 2019). The function “cim” was used to perform QTL analysis following QTL regression model in R (Broman and Sen, 2009). Although, we have validated our QTL analysis results using WinQTLCart 2.5 software (Wang, 2012) employing forward and backward regression of standard model 6 with background marker selection as cofactors. The window size was set at 10 cM, and the walk speed at 1 cM. In this study, composite interval mapping was performed using a p-value (*P* ≥ 0.01) and set a uniform cut-off LOD score value (LOD = 2.5) to identify the number of putative QTLs overall in the analyses, and then we performed a permutation test (1,000 permutations) using a p-value (*P* ≥ 0.05) for each dataset to calculate the LOD significance threshold, which helps in identifying significant QTLs above the threshold for each trait. The LOD scores (LOD ≥ 2.5 in crop plants studies indicate the likelihood ratio of a QTL being present vs. absent. Previously, several crop plant studies have been conducted using LOD ≥ 2.5 for declaring the putative QTLs (Prasad et al., 2003; Kumar et al., 2019, Kumar et al., 2026).

Stable QTLs were defined as QTLs consistently detected across both years of evaluation. QTL mapping was conducted separately for each year, and QTLs with overlapping confidence intervals and consistent additive effects across years were considered the same locus. QTLs explaining ≥10% of PVE were considered major QTLs, whereas those explaining <10% were classified as minor QTLs.

We named the QTLs in this study in the following manner. The QTL name refers to four parts: first part is the sugar QTL, in this case sucrose; the second is the chromosome number where the QTL occurred; the third is the sequence of the QTL; the fourth is the experiment year; For example, “*qSu-Gm05-02*-2018”, *qSu* is the sucrose QTL*; Gm05* is chromosome 5*; 02* is the second QTL*;* 2018 is experiment year. In this study, the candidate genes were identified using SoyBase Genome Browser, (Soybase Browser, n.d., https://www.soybase.org/tools/browsers/gbrowse.html?iframe_pathname_suffix=gmax1.01; https://www.soybase.org/). Significant SNPs were used to identify candidate genes involved in the function and regulation of sugar metabolic pathways using the *Glycine max* genome assembly version Glyma.Wm82.a1 (Gmax1.01).

### Statistical analysis of field trials

A replicated experiment in a RCBD was conducted at the Delta Research and Extension Center (33° 26’ N, -90° 54’ W), Stoneville, MS, in 2017 to increase seed. A RIL population of 201 lines was grown in a two-replication RCBD field trial in 2018 and 2019 at Stoneville, MS, along with parental lines and 12 checks. Analyses of variance (ANOVA) of year, line, and their interactions were conducted in SAS using Proc Mixed (SAS Institute, 2002–2012). Means (maximum and minimum values, and standard deviation (SD) were estimated by Proc Means in SAS (SAS, Statistical Analysis Systems, Cary, NC, USA, 2002–2012) (SAS). Narrow-sense heritabilities were calculated on an entry mean basis according to Holland et al. (2003).

## Results

**Figure 1:**
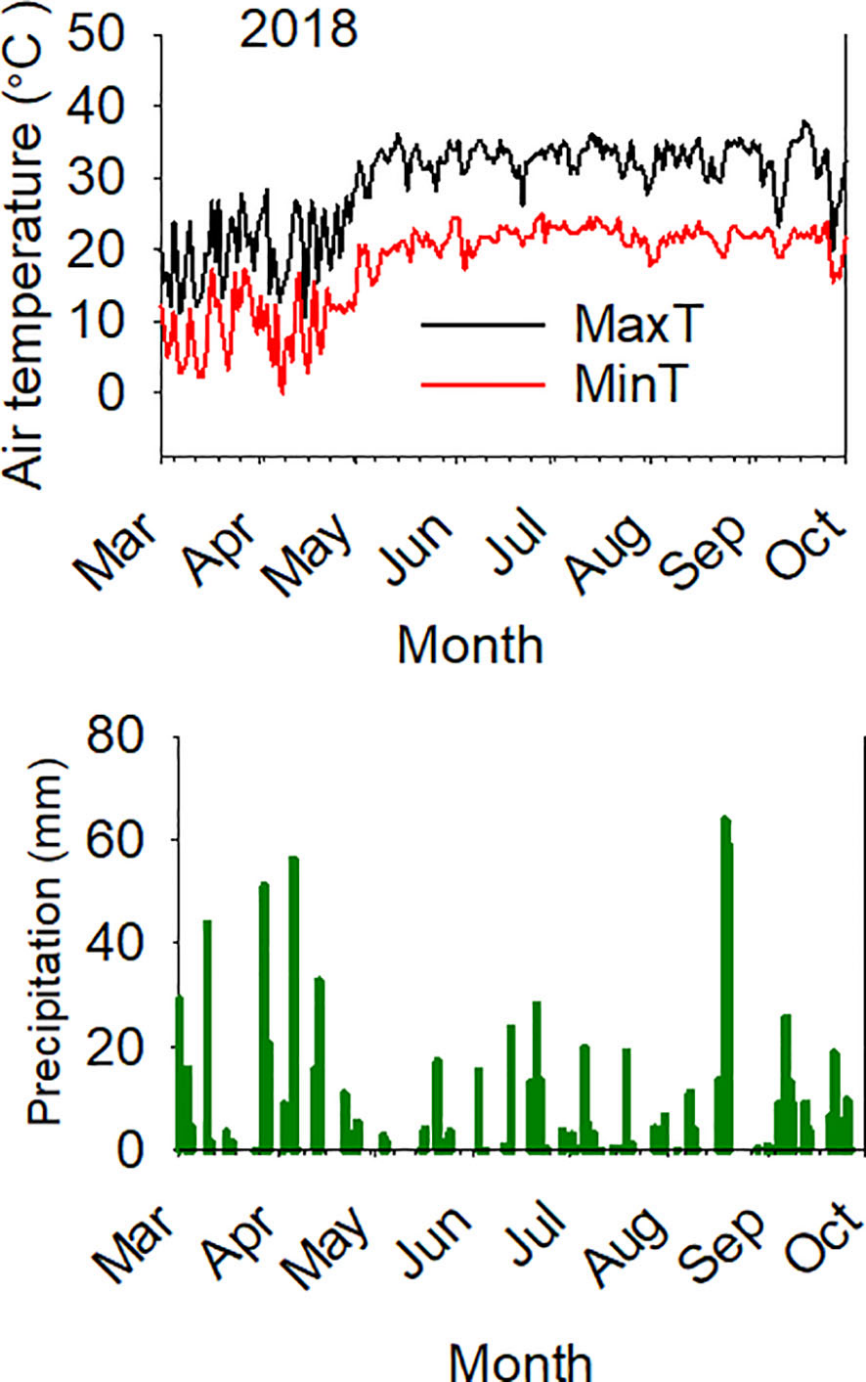
Distribution of daily air temperature (top) and precipitation (bottom) in 2018.Source: http://deltaweather.extension.msstate.edu/weather-station-result/.

**Figure 2:**
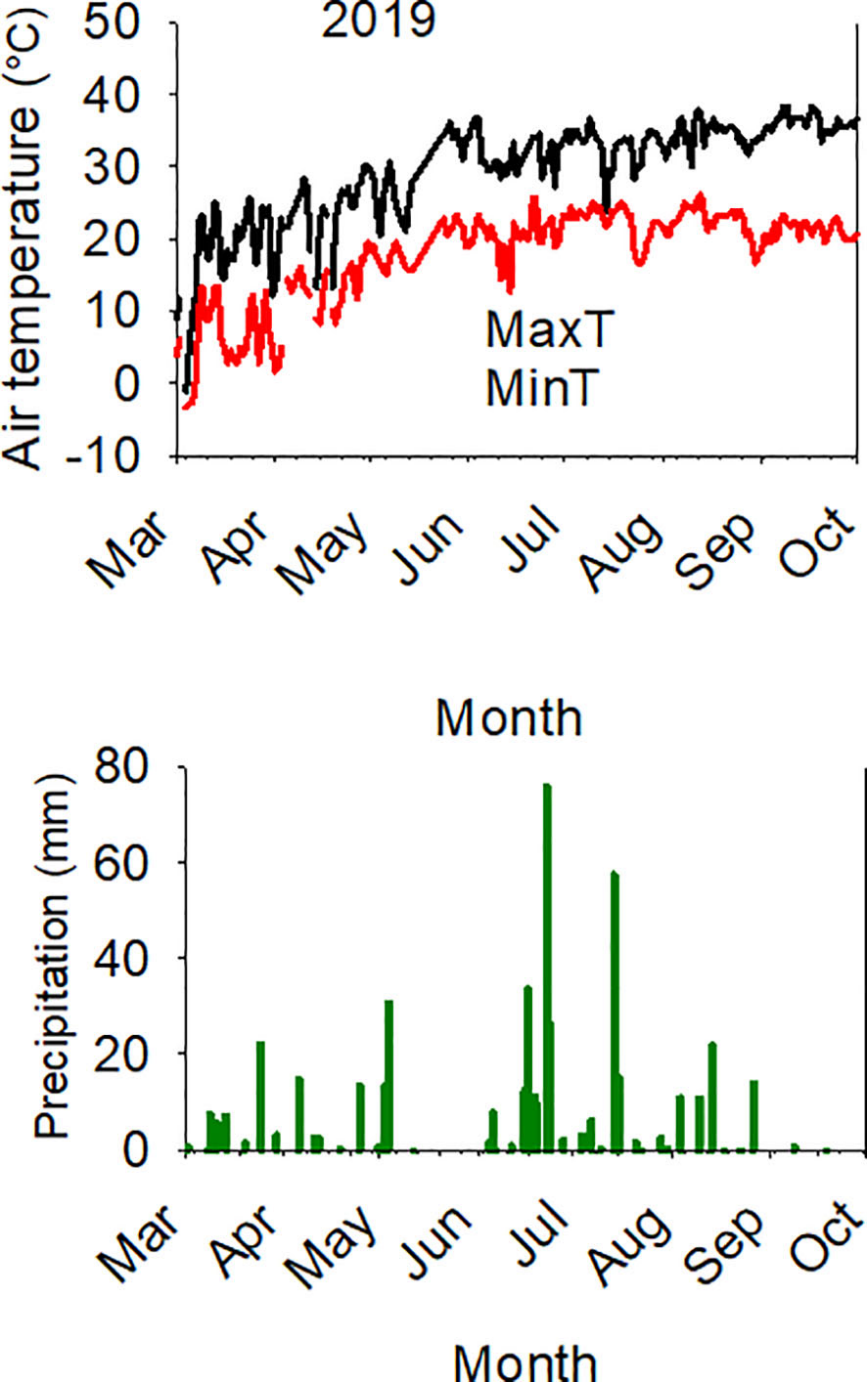
Distribution of daily air temperature (top) and precipitation (bottom) in 2019. Source: http://deltaweather.extension.msstate.edu/weather-station-result/.

ANOVA (**Table 1**) showed that line was highly significant (*P<0.0001*) for raffinose, but not for sucrose or stachyose. Year and year x line interactions were not significant at *P ≤ 0.05* for any sugar. Therefore, the response of sugars in each line was generally the same for sucrose and stachyose, but not so for raffinose (**Table 1**). Within the RIL population, positive correlations between sucrose, raffinose, and stachyose were found in 2018 (**Table 2**), whereas negative correlations between raffinose and sucrose, and between stachyose and raffinose, were found in 2019. Positive correlations between sucrose and stachyose were found in both years. Weather data (**Table 3**; **Figure 1**, **2**) showed different maximum and minimum air temperatures patterns between years, although during June-August, air temperature was only a little higher. It was drier in June and July in 2018 than in 2019; however, the temperature was higher (warmer) and rainfall was lower (drier) in August (drier in 2019 than in 2018) (**Figure 1**, **2**). The narrow sense heritability estimates for sucrose and stachyose were moderate, 0.56 and 0.58, respectively, which indicates that phenotypic selection may be effective for these traits in replicated trials over multiple years.

**Table 1:** Analysis of variance (ANOVA) a for the effects of year, line, and their interactions (year x line) for sucrose, raffinose, and stachyose in the RIL population.

Effect		Sucrose (%)		Raffinose (%)		Stachyose (%)	
	DF	F value	P value	F value	P value	F value	P value
Year	1	0.93	0.34	14.37	0.06	0.36	0.55
line	200	0.98	0.55	1.83	<.0001	1.02	0.44
Year x line	200	0.98	0.54	0.92	0.76	0.96	0.61
Error	402						

*P ≤ 0.05; **P ≤ 0.01; ***P ≤ 0.001.

**Table 2:** Pearson’s correlation coefficients (R and P values) a for sucrose, raffinose, and stachyose in 201 member RIL population in 2018 and 2019.

		2018	2018	2018	2019	2019
Year	Trait	Sucrose	Raffinose	Stachyose	Sucrose	Raffinose
2018	Raffinose	0.31^***^				
	Stachyose	0.71^***^	0.17^*^			
2019	Sucrose	0.51^***^		0.24^***^		
	Raffinose	ns	0.43^***^		-0.03^ns^	
	Stachyose	0.40^***^	ns	0.44^***^	0.59^***^	-0.19^**^

*P ≤ 0.05; **P ≤ 0.01; ***P ≤ 0.001; ns= Nonsignificant.

**Table 3:** Maximum (Max) and minimum (Min) air temperature (T) (°C) and precipitation (mm) in 2018 and 2019.

	2018			2019		
Month	MaxT (°C)	MinT (°C)	Precipitation (mm)	MaxT (°C)	MinT (°C)	Precipitation (mm)
March	19.05	9.05	5.62	16.54	5.36	1.61
April	21.31	9.37	4.56	19.09	8.89	1.24
May	32.08	19.91	1.05	29.36	18.83	2.41
June	32.78	21.91	3.36	31.65	20.37	6.20
July	32.97	22.54	2.18	32.90	22.40	3.11
August	32.85	21.36	5.10	34.32	22.51	1.90
September	31.43	20.96	4.11	36.02	21.20	0.04
October	25.48	14.01	2.33	23.71	12.71	8.83

Source: http://deltaweather.extension.msstate.edu/weather-station-result/.

Data is presented as the average for temperature and total for precipitation.

**Figure 3:**
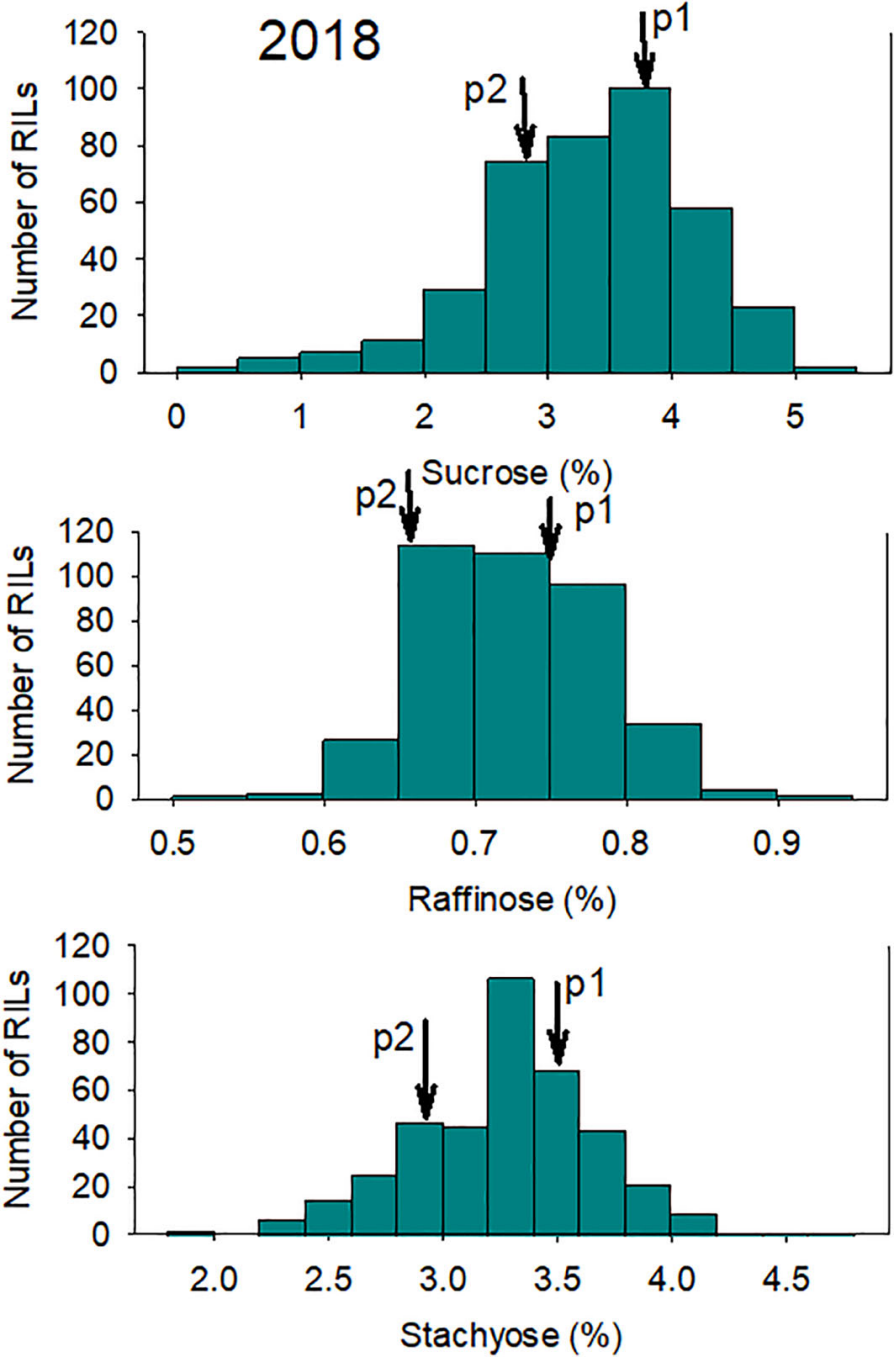
Frequency distribution of sucrose, raffinose, and stachyose in RILs mapping population in 2018. P1= DS25–1 parent; P2= DT97-4290 parent.

**Figure 4:**
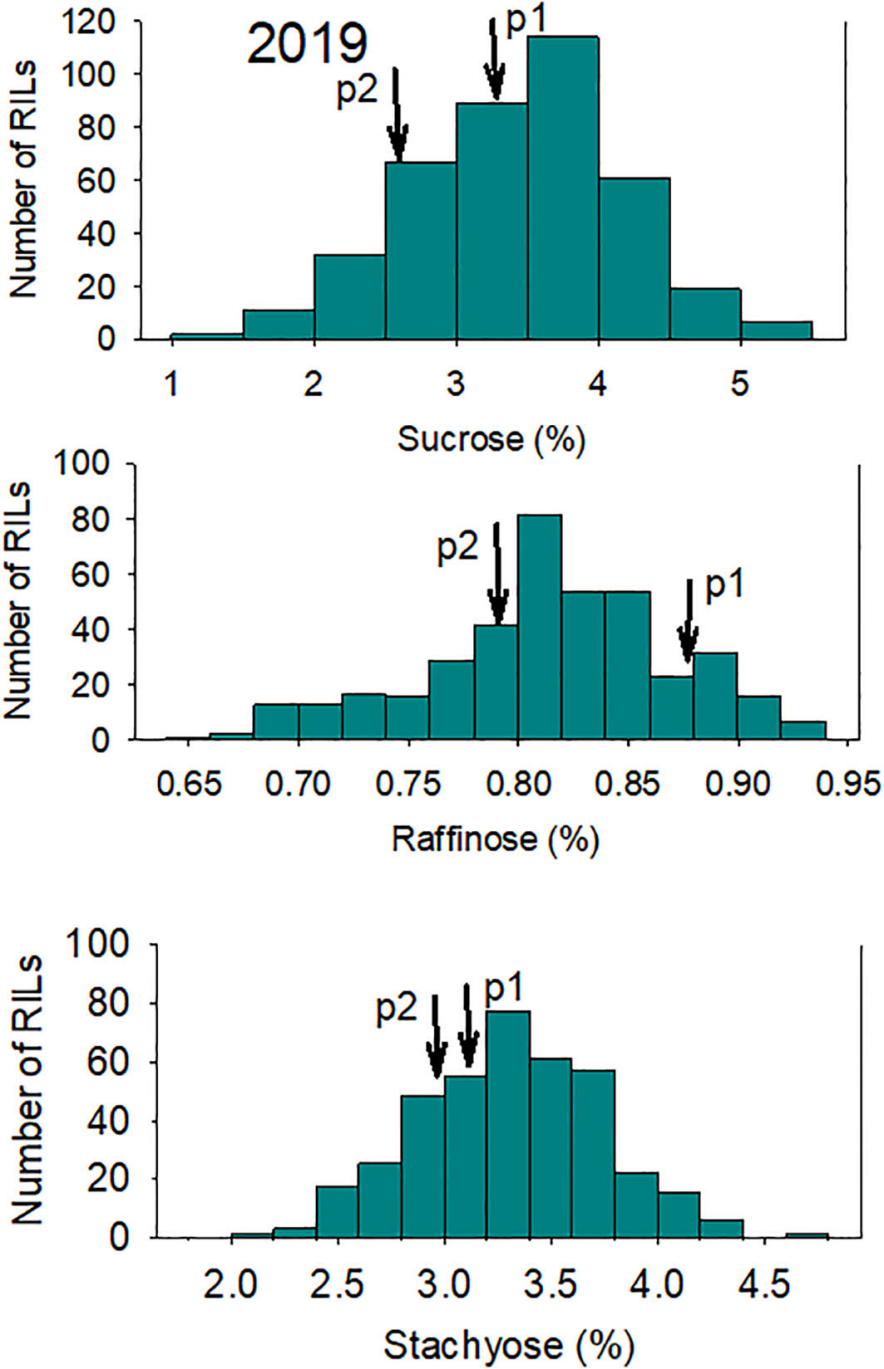
Frequency distribution of sucrose, raffinose, and stachyose in RILs mapping population in 2019. P1= DS25–1 parent; P2= DT97–4290 parent.

The distribution of sugar content (**Figure 3**, **4**, **Table 4**. **Supplementary Table 1**) showed normal distributions, but the wide range of nutrients concentrations in the RILs reflect the physiological and genetic complexities and their interactions with the traits. The interaction of physiological and genetic factors with environmental factors such as temperature and rainfall resulted in some genotypes accumulating more nutrients than others (Marschner, 2012; Bellaloui et al., 2010).

**Table 4:** The Means procedure in SAS to obtain means, minimum, and maximum values (%), and confidence limit (CL) at 95%, and standard deviation (SD) for sucrose, raffinose, and stachyose in the 201 member RIL population.

			2018				
Variable	Mean	Minimum	Maximum	Median	Lower 95%	Upper 95%	SD
					CL for mean	CL for mean	
Sucrose (%)	3.32	1.60	5.10	3.40	3.24	3.41	0.85
Raffinose (%)	0.72	0.53	0.94	0.72	0.72	0.73	0.057
Stachyose (%)	3.28	1.80	4.80	3.30	3.24	3.32	0.41
							
			2019				
Variable	Mean	Minimum	Maximum	Median	Lower 95%	Upper 95%	SD
					CL for mean	CL for mean	
Sucrose (%)	3.43	1.30	5.50	3.50	3.36	3.50	0.75
Raffinose (%)	0.82	0.70	0.90	0.80	0.81	0.83	0.06
Stachyose (%)	3.31	1.90	4.80	3.30	3.27	3.36	0.45

In 2018, a total of four QTL was identified for sucrose (**Table 5**). These four QTL (*qSu-Gm03-01*-2018, *qSu-Gm05-02*-2018, *qSu-Gm11-03*-2018, and *qSu-Gm19-04*-2018) were distributed on chrs 3, 5, 11, and 19 at the positions 34.46, 67.70, 88.06, and 132.74 cM, with LODs ranging from 2.57 to 3.89 (**Table 5**), while the threshold LOD score for sucrose 2018 was 3.85 after permutations. These QTL accounted for 5.7% to 6.56% of the phenotypic variation for sucrose. Two QTL (*qRaf-Gm06-01–*2018 and *qRaf-Gm20-02*-2018) were detected for raffinose in 2018 on chrs 6 and 20, positioned at 48.28 and 120.47 cM, with LODs of 3.59 and 3.77, and explained 5.78% and 8.62% of the phenotypic variation, respectively. The threshold LOD score for raffinose 2018 was 3.75. Two QTL were identified for stachyose (*qSta-Gm06-01–*2018 and *qSta-Gm19-02*-2018) on Chrs 6 and 19, positioned at 130.64 and 67.38 cM, with LODs of 3.11 and 2.74, and explained 7.56% and 4.77% of the phenotypic variation, respectively, although the threshold LOD score for stachyose 2018 was 3.88.

**Table 5:** Novel and previously reported significant QTL (with LOD ≥ 2.5) identified in the 201 member RIL population using interval mapping in R/qtl.

Trait	QTL	Closest SNP (bp) Marker	Chromosome /linkage group	Position (cM)	LOD	% PVE	Additive Effect	Reference
Sucrose-2018	*qSu-*Gm03*-01*-2018	*S03_36257859*	Gm03/Lg1_N	88.06	3.32	5.70	0.18	Hu et al., 2023
	*qSu-*Gm05*-02*-2018	*S05_42036315*	Gm05/Lg1_A1	132.74	3.38	5.53	-0.18	Novel
	*qSu-*Gm11*-03*-2018	*S11_4951621*	Gm11/Lg1_B1	34.46	3.89	7.27	-0.20	Novel
	*qSu-*Gm19*-04*-2018	*S19_43072284*	Gm19/Lg1_L	67.70	2.57	6.56	-0.19	Novel
Sucrose-2019	*qSu-Gm05-01*-2019	*S05_42036315*	Gm05/Lg1_A1	132.74	4.83	6.29	-0.19	Novel
	*qSu-Gm09-02*-2019	*S09_47467654*	Gm09/Lg2_K	32.60	2.89	3.65	0.14	SoyBase This QTL overlapped within a QTL reported at 27.5- 37.43 cM.
	*qSu-Gm17-03*-2019	*S17_18296645*	Gm17/LG/D2	100.03	3.28	2.04	-0.11	Novel
	*qSu-*Gm19*-04*-2019	*S19_39486942*	Gm19/Lg1_L	45.58	3.44	0.43	-0.05	Novel
Raffinose_2018	*qRaf-*Gm06*-01*-2018	*S06_19595695*	Gm06/Lg1_C2	120.47	3.59	5.78	-0.01	SoyBase This QTL is 2.3 Mb apart from SNP marker reported at 17.2 Mb.
	*qRaf-*Gm20*-02*-2018	*S20_35185358*	Gm20/Lg1_I	48.28	3.77	8.62	-0.01	Novel
Raffinose_2019	*qRaf-*Gm06*-01*-2019	*S06_18481138*	Gm06/Lg1_C2	116.73	7.88	15.38	-0.01	SoyBase This QTL is 1 Mb apart from SNP marker reported at 17.2 Mb.
	*qRaf-*Gm14*-02*-2019	*S14_46549574*	Gm14/Lg1B2	118.32	2.75	5.10	-0.01	Novel
	*qRaf-*Gm19*-03*-2019	*S19_45757122*	Gm19/Lg1_L	90.89	3.64	5.73	0.01	Novel
	*qRaf-*Gm20*-04*-2019	*S20_35243107*	Gm20/Lg1_I	48.69	4.35	6.25	0.01	Novel
Stachyose_2018	*qSta-*Gm06*-01*-2018	*S06_43979515*	Gm06/Lg1_C2	130.64	3.11	7.56	-0.09	Hu et al., 2023 This QTL is 0.5 Mb a part of QTL at 44.6 Mb.
	*qSta-*Gm19*-02*-2018	*S19_43028773*	Gm19/Lg1_L	67.38	2.74	4.77	-0.07	Knizia et al., 2023 This QTL also reported by SoyBase at 65 to 74 cM.
Stachyose_2019	*qSta-*Gm03*-01*-2019	*S03_35672657*	Gm03/Lg1_N	85.14	3.81	4.96	0.08	Novel
	*qSta-*Gm13*-02*-2019	*S13_27940207*	Gm13/Lg1_F	68.28	3.88	7.48	-0.09	Novel
	*qSta-*Gm19*-03*-2019	*S19_47196014*	Gm19/Lg1_L	103.54	2.50	3.26	-0.06	Knizia et al., 2023 This QTL co-localized between two QTLs at 37–43 Mb and 49 to 50 Mb.

In 2019, 4 QTL (*qSu-Gm05-01*-2019, *qSu-Gm09-02*-2019, *qSu-Gm17-03*-2019, and *qSu-Gm19-04*-2019) were identified on chrs 5, 9, 17, 19, and positioned at 132.74, 32.60, 100.03, and 45.58 cM, respectively with LODs ranging from 2.89 to 4.83, and explaining 0.43 to 6.29% of the phenotypic variation for sucrose. Four QTL (*qRaf-Gm06-01*-2019, *qRaf-Gm14-02*-2019, *qRaf-Gm19-03*-2019, *qRaf-Gm20-04*-2019) were detected for raffinose on chrs 6, 14, 19, and 20), positioned at 116.73, 118.32, 90.89, and 48.69 cM, with LODs ranging from 2.75 to 7.88. The threshold LOD score for raffinose 2019 was 3.79. These QTL accounted for 5.10% to 15.38% of the phenotypic variation for raffinose. Three QTL (*qSta-Gm03-01*-2019, *qSta-Gm13-02*-2019, *qSta-Gm13-02*-2019) were identified for stachyose on chrs 3, 13, 19, respectively, positioned at 85.14, 68.28, and 103.54 cM, with LODs ranging from 2.50 to 3.88, and explaining 3.26 to 7.48% of the phenotypic variability of stachyose. The threshold LOD score for stachyose 2019 was 3.77. Positive and negative additive effects were observed for sucrose, raffinose, and stachyose in each year, except for sucrose and raffinose in 2019, where only positive additive effects were noticed (**Table 5**). A search of soybase and the literature indicated that 12 QTL (was not previously reported; therefore, they are novel (**Table 5**). These novel QTLs are: *qSu-Gm05-02-2018, qSu-Gm11-03-2018, qSu-Gm19-04-2018, qSu-Gm05-01-2019, qSu-Gm17-03-2019, qSu-Gm19-04-2019, qRaf-Gm20-02-2018, qRaf-Gm14-02-2019, qRaf-Gm19-03-2019, qRaf-Gm20-04-2019, qSta-Gm03-01-2019, and qSta-Gm13-02-2019.*

## Discussion

**Figure 5:**
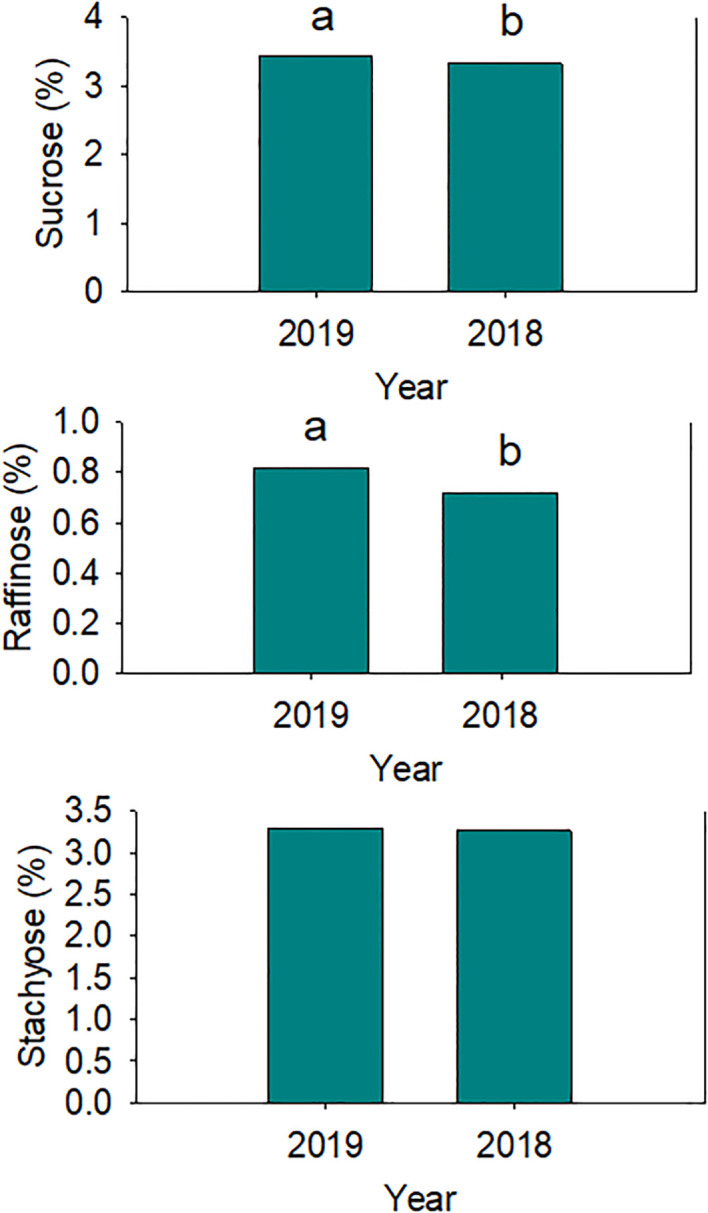
Sucrose, raffinose, and stachyose across RILs in 2018 and 2019. No letters on stachyose bars means there is no significant differences between years.

The weather differences during our experiments were reflected by the pattern of the daily air temperatures and precipitation (**Figure 1**, **2**). It is clear that the lines did not experience a uniform environment during seed-fill and/or at maturity. These differences in temperature and rainfall could have affected some seed sugar contents such as raffinose, although some other sugars such as sucrose and stachyose were not significantly affected as these weather components may not have been different enough to cause any sugar differences in sucrose and stachyose. Although the ranking of lines for sugars did not change between years, as the interaction of line × year was not significant, seed sucrose and raffinose contents were higher in 2019 than in 2018 (**Figure 5**). This indicated that sucrose and raffinose may be more responsive to temperature and drought changes than stachyose (**Figure 5**). The positive and negative correlations between seed sugars in this study have also been observed in other soybean seed nutrients. For example, previous research showed that temperature resulted in different trends (positive or negative correlations) in seed composition components, including seed protein, oil, sugars, and minerals (Bellaloui et al., 2009, Bellaloui et al., 2010, Bellaloui et al., 2011). Differences in weather patterns within and across years, plus the limited number of environments (2018 and 2019) and replications within years (two) may have contributed to some QTL inconsistencies across years.

For sucrose, QTLs *qSu-Gm05-02–*2018 and *qSu-Gm05-01–*2019 were identified on the same chromosome (*Gm05*) in both 2018 and 2019, and also found at the same position (132.74 cM), with LODs of 3.38 and 4.83, respectively. The other two sucrose QTL, *qSu-Gm19-04–*2018 and *qSu-Gm-19-04*-2019, were identified on the same chromosome in 2018 and in 2019, but at different positions (67.70 and 45.58 cM), with LODs of 2.57 and 3.44, respectively. Similar observations were noticed for raffinose QTL *qRaf-Gm06-01–*2018 and *qRaf-Gm06-01–*2019 in 2018 and 2019 (at positions of 120.47 and 116.73cM, respectively); for raffinose QTL *qRaf-Gm20-02–*2018 and *qRaf-Gm20-04–*2019 at positions 48.28 and 48.69 cM with LODs of 3.77 and 4.35, respectively, and for stachyose QTL *qSta-Gm19-02–*2018 and *qSta-Gm19-03–*2019 in 2018 and 2019 positioned at 67.38 and 103.54 cM with LODs of 2.74 and 2.50, respectively.

QTL *qSu-Gm05-02–*2018 for sucrose with the marker *S05_42036315* at position 132.74 cM occurred in both years and at the same position on the same chr (5). The same observation was noticed for QTL *qRaf-Gm20-02–*2018 with SNP markers *S20_35185358* in 2018 and *S20_35243107* in 2019 at positions 48.28 and 48.69 cM, respectively, where this QTL occurred each year at essentially the same position. For sucrose and raffinose, the above two QTL were consistently detected in 2018 and 2019, which can be considered stable QTL across years.

The same may be true for other QTL even though the position is slightly different across years; for example, QTL *qSu-Gm19-04–*2018 with SNP marker *S19_43072284* in 2018 and *S19_39486942* in 2019, positioned at 67.70 cm in 2018, and at 45.58 cM in 2019; similarly for *qRaf-Gm06-01–*2018 at 120.47 cM in 2018 and *qRaf-Gm06-01–*2019 at 116.73 cM in 2019, and similarly for *qSta-Gm19-02–*2018 at 67.38 cM and *qSta-Gm19-03–*2019 at 103.54 cM in 2018 and 2019, respectively. Therefore, the QTL *qSu-Gm19-04–*2018 and *qSu-Gm19-04*-2019; *qRaf-Gm06-01–*2018 and *qRaf-Gm06-01*-2019, and *qSta-Gm19-02–*2018 and *qSta-Gm19-03*-2019, are three QTL that were repeated in each year, may reflect significance and stability across years, and may potentially be used for sugar profiling to select for desirable sugars. The disparity of chromosome locations across years for the three above comparisons may likely have been much less if more environments and replications had been used. Hence, additional testing will be required to stabilize these QTL before they can effectively be used as markers in breeding.

**Figure 6:**
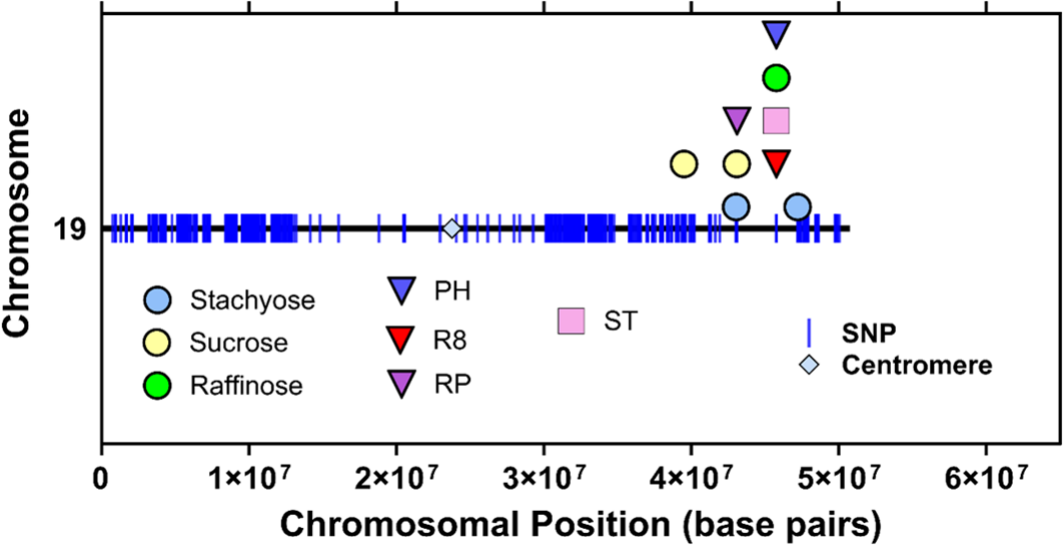
QTLs associated with stachyose, sucrose, raffinose, plant height (PH), maturity (R8), reproductive period (RP), and stem termination (ST) on chromosome 19.

The occurrence of the following QTLs, below, in 2018 and 2019 on Chrs 19 and 6 may indicate the stability of these QTLS and the significance of these chromosomes to harbor several important QTLs related to other traits: *qSu-Gm19-04–*2018 and *qSu-Gm19-04–*2019 for sucrose; QTLs *qRaf-Gm06-01–*2018 and *qRaf-Gm06-01–*2019 for raffinose; QTLs *qRaf-Gm20-02–*2018 and *qRaf-Gm20-04–*2019 for raffinose; and QTLs *qSta-Gm19-02–*2018 and *qSta-Gm19-03–*2019 for stachyose. Our search revealed that Chr. 19 harbors several QTLs related to other traits (**Figure 6**). Further research is needed to identify more QTLs, molecular markers, and genes.

Searching SoyBase revealed there are only 31 QTL associated with seed sucrose, and 15 of these QTL were associated with oligosaccharides, including raffinose and stachyose (https://legacy.soybase.org/search/index.php?searchterm=raffinose+qtl&list=bi_parental_qtl_ontology_listview; https://legacy.soybase.org/). All QTLs reported in SoyBase are reported here in this study. For example, Kim et al. (2005), using 117 F_2:10_ recombinant inbred lines, developed from a cross of ‘‘Keunolkong’’ and ‘‘Shinpaldalkong’’, were able to identify 4 QTL for oligosaccharide content on linkage groups (LG) C2 (chr 2), H (chr 12), J (chr 16), and L (chr 19), and 2 QTL were associated with sucrose content on LG H (chr 12) and J (chr 16). Also, they were able to detect 2 QTL for the total oligosaccharides and sucrose content on LG H (chr 12) and J (chr 16). Zeng et al. (2014), used simple sequence repeats (SSR) and SNPs in an F_2_–derived QTL mapping population, which was a cross between a low sucrose line, MFS-553, and a high sucrose plant introduction, PI243545. Among 626 SSR primers used, they found 209 SSR markers were polymorphic. The polymorphic SSR markers were used to genotype 220 F_2:3_ lines. The resulting population of 94 F_3:4_ lines derived from the initial F_2:3_ population was genotyped with 5,361 SNP markers, among which 2,016 were polymorphic. They were able to identify 3 novel QTLs for seed sucrose on chromosomes 5, 9, and 16, accounting for 46, 10 and 8%, respectively, of the phenotypic variation for sucrose content. They concluded that SSR and SNP markers linked to these QTLs can be used for marker-assisted selection (MAS) to select for the desired sugar profile. Salari et al. (2021), used 140 SoyNAM (Nested Association Mapping) RILs, from a cross between IA3023 and LD02-4485, and 3,038 SNP markers from the Illumina SoyNAM BeadChip SNP, to map the QTL for sucrose, raffinose and stachyose. They were able to identify 3 QTLs for sucrose, one on chr 1, and 2 QTL on chr 3. These QTL explained 10% and 22% of the phenotypic variability, respectively. Also, they detected 1 QTL for raffinose on chr 6, which accounted for 6% of the phenotypic variability, and postulated that these QTL harbor candidate genes involved in plant growth and seed development.

Recently, Knizia et al. (2023) used a ‘Forrest’ by ‘Williams 82’ RIL soybean population with 309 lines, to identify a total of 15 QTL controlling seed sugar content; 3 QTL for raffinose (two on chr 9, and one on chr 12); and 7 QTL for stachyose on chr 9, 12, 13, 16, 17, and 20. They were also able to identify 57 candidate genes, among which were a cluster of four genes involved in the sugar pathway on chr 17. Hu et al. (2023) identified 72 QTL, among which 19 QTL were for sucrose, 9 QTL for raffinose, and 11 for stachyose. Liu et al. (2023) detected 11 QTL for sucrose on chr 6, 7, 9, 13, 17, 19, and 20, and were also able to identify 233 candidate genes that were involved in sugar pathways and metabolism. Two QTL for sucrose on chr 14 and 11 were identified by Jamison et al. (2024), one QTL of which for sucrose was clustered with QTL for protein and oil content, reflecting the complexity of seed composition inheritance as a quantitative trait.

In our current study, a total of 19 QTL, among which 12 were novel, were identified, as shown in **Table 5**. In 2018, a total of 8 QTL was identified for sugars (4 QTL for sucrose on chrs 3, 5, 11, and 19; 2 QTL for raffinose on chrs 6 and 20; and 2 QTL for stachyose on Chrs 6 and 19). In 2019, 4 QTL for sucrose on chrs 5, 9, 17, 19; 4 QTL for raffinose on chrs 6, 14, 19, and 20; and 3 QTL for stachyose on chrs 3, 13, 19. It is important to note that QTL *qSu-Gm05-02–*2018 for sucrose with the marker *S05_42036315* at position 132.74 cM occurred in both years and at the same position on the same chr (5); and the same observation was noticed for QTL *qRaf-Gm20-01–*2018 with SNP marker *S20_35185358* in 2018 and *S20_35243107* in 2019 at position 48 cM, where this QTL occurred in each year at essentially the same position. These two QTL are important as they occurred across only two years, indicating their strong stability and possible use by breeders for sugar selection.

Although some QTL identified here were also reported elsewhere on the same chromosomes (chr 3, 5, 6, 11, 14, 19, and 20) (Maughan et al., 2000; Kim et al., 2005, Kim et al., 2006; Skoneczka et al., 2009; Wang et al., 2014; Akond et al., 2015; Xu et al., 2022; Liu et al., 2023), the QTL identified in the current study used a unique RIL population that is also segregating for heat-tolerance, perhaps providing unique QTL that can help breeders to select for desirable traits, such as seed heat tolerance, as well as against undesirable seed traits, such as raffinose and stachyose under high heat growing conditions.

Although the overall line effect was not statistically significant for seed sucrose and stachyose at the population mean level, this does not necessarily indicate a lack of genetic variation within the RIL population. ANOVA evaluates differences among line means averaged across the genome, whereas QTL mapping detects associations between specific genomic regions and phenotypic variation. Thus, loci with moderate or small effects can be identified even when overall line differences are not statistically significant. Importantly, the two parental lines exhibited statistically significant differences for all evaluated traits (**Figure 3**, **4**), confirming the presence of contrasting alleles and sufficient genetic variation segregating in the RIL population. Increasing the number of replicates can reduce the environmental noise and enhance the statistical power to detect significant differences, however; evaluating (n ≈ 201) RILs would require substantial additional resources. Nevertheless, the RIL population captures recombination events that partition parental alleles into different combinations across lines, enabling the detection of genomic regions associated with trait variation.

In this study, only one locus showed relatively large effects for Raffinose (>10% PVE), whereas most QTLs had modest contributions to trait variation. This pattern is consistent with the complex genetic architecture of seed sugar composition, which is regulated by multiple genes involved in carbohydrate metabolism and transport pathways. Consequently, the predominance of small-effect QTLs observed here likely reflects the polygenic control of these traits rather than limitations of the mapping approach.

From a breeding perspective, major QTLs may provide immediate targets for marker-assisted selection due to their larger and more predictable effects on phenotype. However, minor QTLs also have practical value because the cumulative effect of multiple small-effect loci can substantially influence seed sugar composition. Interestingly, some minor QTL identified in this study showed relatively large additive effects despite explaining a small proportion of phenotypic variance. This reflects the influence of allele frequency and environmental variance on PVE estimates. Such loci may still be valuable for breeding if favorable alleles can be fixed through selection. In addition, the identification of stable QTLs across years is particularly important, as these loci are less likely to be influenced by environmental variation and therefore represent more reliable targets for selection. Improvement of seed sugar traits will likely require pyramiding favorable alleles from several loci or the application of genomic selection strategies that capture the combined effects of many small-effect QTLs.

In addition, because limited information is available on the genetic control of seed sugar traits, QTL mapping was conducted separately for each year to capture temporal variation and identify loci potentially influenced by environmental conditions. This approach provides important baseline insight into the genetic architecture of these understudied traits. Although combined analysis across years can improve detection of stable QTLs, the year-specific analyses performed here contribute to understanding the mechanisms underlying seed sugar accumulation and highlight loci that warrant further validation in multi-environment trials.

Several QTL identified in the present study were detected in both 2018 and 2019 on the same chromosomes and at similar genomic positions, suggesting temporal repeatability. Notably, QTL *qSu-Gm05-02–2018* and *qSu-Gm05-01–2019* for sucrose, as well as *qRaf-Gm06-01–2018* and *qRaf-Gm06-01–2019* for raffinose, were mapped to comparable positions across years, indicating consistent genetic control. Additional QTL pairs detected on chromosomes 19 and 6 for sucrose, raffinose, and stachyose showed minor positional shifts between years, which may reflect environmental influences on QTL expression rather than different loci. The repeated detection of these regions highlights chromosomes 19 and 6 as important genomic regions harboring genes involved in seed sugar composition.

Searches of SoyBase (https://legacy.soybase.org/search/index.php?searchterm=raffinose+qtl&list=bi_parental_qtl_ontology_listview; https://legacy.soybase.org/) indicate that relatively few QTL associated with seed sugars have been reported to date, with approximately 31 QTL linked to sucrose and 15 associated with oligosaccharides, including raffinose and stachyose. Earlier studies identified QTL on several chromosomes using diverse populations and marker systems. For example, Kim et al. (2005) detected QTL for oligosaccharides on linkage groups corresponding to chromosomes 2, 12, 16, and 19, as well as QTL for sucrose on chromosomes 12 and 16. Using SSR and SNP markers, Zeng et al. (2014) identified three novel QTL for sucrose on chromosomes 5, 9, and 16 that explained substantial phenotypic variation. Salari et al. (2021), working with a SoyNAM population, reported QTL for sucrose on chromosomes 1 and 3 and a raffinose QTL on chromosome 6. More recently, Knizia et al. (2023) identified multiple QTL for raffinose and stachyose across several chromosomes using a Forrest × Williams 82 population, while Hu et al. (2023) and Liu et al. (2023) detected numerous QTL and candidate genes involved in sugar metabolism pathways. Jamison et al. (2024) further demonstrated that QTL for sucrose may co-localize with loci controlling protein and oil content, highlighting the complex genetic regulation of seed composition traits.

In the current study, a total of 19 QTL was identified across two years, of which 12 appear to be novel relative to previously reported loci. Several QTL overlapped with genomic regions previously associated with sugar traits on chromosomes 3, 5, 6, 11, 14, 19, and 20 (Magham et al., 2000; Kim et al., 2005, Kim et al., 2006; Skoneczka et al., 2009; Wang et al., 2014; Akond et al., 2015; Xu et al., 2022; Liu et al., 2023), while others were unique to this population. The use of a RIL developed for heat tolerance likely contributed to the identification of distinct QTL, suggesting potential genetic mechanisms underlying seed sugar composition under high-temperature conditions.

Genes located within ±0.3 Mb of significant SNPs associated with sucrose, raffinose, and stachyose were considered potential candidates. This search identified more than 50 genes within QTL intervals involved in sugar metabolism, transport, regulatory processes, and stress responses (**Table 6**, **Supplementary Table 2**; **Supplementary Figure 2**). Notably, several loci contained genes directly related to carbohydrate pathways, including sugar kinase, glycosyl-/glucuronosyl transferases, fructose-bisphosphate aldolase, and galactinol synthase-related proteins, which are key components of sucrose utilization and raffinose family oligosaccharide biosynthesis. QTL regions also harbored regulatory and stress-responsive genes such as heat shock proteins, drought-induced proteins (DI19), auxin/IAA-related genes, transcription factors, and metal transporters, suggesting that sugar accumulation may be influenced by both metabolic processes and environmental adaptation mechanisms.

**Table 6:** Candidate genes a for sucrose, raffinose, and stachyose using DS25-1 × DT97–4290 RIL population.

QTL	Name	Location	Gene annotation/gene ontology/functional genomics
*qSu-Gm03-01-2018*	Glyma03g28120	Gm03:35969569.35972058	10 KDA HEAT SHOCK PROTEIN
	Glyma03g28410	Gm03:36294501.36322871	GLUTAMATE SYNTHASE
*qSu-Gm05-02-2018*	Glyma05g38540	Gm05:41869789.41875270	AUXIN RESPONSE FACTOR
	Glyma05g38510	Gm05:41844917.41850967	ATP-DEPENDENT CLP PROTEASE
*qSu-Gm11-03-2018*	Glyma11g06640	Gm11:4684961.4693122	TRANSCRIPTION FACTOR MEIS1 AND RELATED HOX DOMAIN PROTEINS
	Glyma11g06880	Gm11:4850212.4852030	UDP-GLUCOSYLTRANSFERASE
*qSu-Gm19-04-2018*	Glyma19g35236	Gm19:42778315.42799255	ATP-BINDING CASSETTE TRANSPORTER
	Glyma19g35401	Gm19:42933164.42936374	SUGAR KINASE
*qRaf-Gm06-01-2018*	Glyma06g22730	Gm06:19469580.19474113	GLYCOSYLTRANSFERASE 8 DOMAIN-CONTAINING PROTEIN
	Glyma06g22820	Gm06:19708865.19710648	GLUCOSYL/GLUCURONOSYL TRANSFERASES
	Glyma06g22812	Gm06:19674533.19678514	GDP-FUCOSE PROTEIN O-FUCOSYLTRANSFERASE
	Glyma06g22812	Gm06:19674533.19678514	GDP-FUCOSE PROTEIN O-FUCOSYLTRANSFERASE
*qRaf-Gm20-02-2018*	Glyma20g25170	Gm20:34912898.34913895	ZINC FINGER PROTEIN WITH KRAB AND SCAN DOMAINS
	Glyma20g25580	Gm20:35243672.35245204	AUX/IAA FAMILY
	Glyma20g25712	Gm20:35343272.35347269	CCCH ZINC FINGER/TIS11-RELATED
	Glyma20g25790	Gm20:35429047.35431858	FRUCTOSE-BISPHOSPHATE ALDOLASE
	Glyma20g25800	Gm20:35432475.35451008	SUBFAMILY NOT NAMED); ATP-DEPENDENT RNA HELICASE A
*qRaf-Gm06-01-2019*	Glyma06g21730	Gm06:18295701.18298404	DESCRIPTION UNAVAILABLE)); C2H2-LIKE ZINC FINGER PROTEIN
	Glyma06g22030	Gm06:18759337.18760652	GLYCOSYL HYDROLASES FAMILY 28
*qRaf-Gm14-02-2019*	Glyma14g37170	Gm14:46444640.46446280	UDP-GLUCOSYLTRANSFERASE
	Glyma14g37260	Gm14:46546240.46549920	SUGAR KINASE
	Glyma14g37522	Gm14:46817720.46829863	FRUCTOSE-2,6-BISPHOSPHATASE
	Glyma14g37560	Gm14:46842224.46845706	ZINC/IRON TRANSPORTER, PLANT AND YEAST
	Glyma14g37540	Gm14:46835726.46836988	NITRATE, FROMATE, IRON DEHYDROGENASE
*qRaf-Gm19-03-2019*	Glyma19g38854	Gm19:45715838.45717982	FOLATE SYNTHESIS PROTEINS
*qRaf-Gm20-04-2019*	Glyma20g25580	Gm20:35243672.35245204	AUX/IAA FAMILY
	Glyma20g25640	Gm20:35284346.35291382	LIPID PHOSPHATE PHOSPHATASE
	Glyma20g25580	Gm20:35243672.35245204	AUX/IAA FAMILY
	Glyma20g25530	Gm20:35194535.35196239	RETICULON-RELATED (PLANT
	Glyma20g25660	Gm20:35302560.35307176	AMINO ACID PERMEASE-RELATED
*qSta-Gm06-01-2018*	Glyma06g40601	Gm06:43773270.43776039	LEUCINE-RICH REPEAT RECEPTOR-LIKE PROTEIN KINASE
	Glyma06g40860	Gm06:44086613.44089781	GH3 AUXIN-RESPONSIVE PROMOTER
	Glyma06g40880	Gm06:44109515.44112977	LEUCINE-RICH REPEAT RECEPTOR-LIKE PROTEIN KINASE
*qSta-Gm02-2018*	Glyma19g35251	Gm19:42786701.42790760	ATP-BINDING CASSETTE TRANSPORTER (PDR)
	Glyma19g35401	Gm19:42933164.42936374	SUGAR KINASE
	Glyma19g35560	Gm19:43115295.43118308	HEAT SHOCK PROTEIN 70KDA
	Glyma19g35670	Gm19:43166744.43169591	SUBFAMILY NOT NAMED); PF00076 (RNA RECOGNITION MOTIF. (A.K.A. RRM, RBD, OR RNP DOMAIN))
*qSta-Gm19-01-2019*	Glyma03g27770	Gm03:35564260.35566476	CYTOCHROME P450 CYP4/CYP19/CYP26 SUBFAMILIES
	Glyma03g27790	Gm03:35570186.35581178	UBIQUITIN C-TERMINAL HYDROLASE
	Glyma03g27800	Gm03:35582912.35588047	H+/OLIGOPEPTIDE SYMPORTER);
	Glyma03g27830	Gm03:35604981.35613864	OLIGOPEPTIDE TRANSPORTER-RELATED
	Glyma03g27840	Gm03:35616234.35623127	H+/OLIGOPEPTIDE SYMPORTER
	Glyma03g27865	Gm03:35640069.35643599	TRANSCRIPTION FACTOR
*qSta-Gm13-02-2019*	Glyma13g24260	Gm13:27635616.27643692	GUANYLATE BINDING PROTEIN
	Glyma13g24440	Gm13:27839025.27840078	HEAT-SHOCK PROTEIN 17); MOLECULAR CHAPERONE; SMALL HEAT-SHOCK PROTEIN HSP26/HSP42
	Glyma13g24440	Gm13:27839025.27840078	HEAT-SHOCK PROTEIN 17
	Glyma13g24461	Gm13:27847776.27854806	HEAT-SHOCK PROTEIN 17
	Glyma13g24420	Gm13:27830405.27834313	DROUGHT INDUCED 19 PROTEIN (DI19), ZINC-BINDING
	Glyma13g24420	Gm13:27830405.27834313	PF05605 (DROUGHT INDUCED 19 PROTEIN (DI19), ZINC-BINDING; AT3G05700.1 (DROUGHT-RESPONSIVE FAMILY PROTEIN
	Glyma13g24470	Gm13:27850355.27853113	UBIQUITIN); KOG0005 (UBIQUITIN-LIKE PROTEIN
	Glyma13g24860	Gm13:28171233.28173685	HEAT SHOCK TRANSCRIPTION FACTOR
*qSta-Gm19-02-2019*	Glyma19g40550	Gm19:46915407.46918937	RAFFINOSE SYNTHASE OR SEED IMBIBITION PROTEIN SIP1
	Glyma19g40680	Gm19:47029812.47032065	GALACTINOL SYNTHASE-RELATED
	Glyma19g40740	Gm19:47080241.47083418	GLYCOSYL HYDROLASES FAMILY 28

^a^ The above gene annotation/functional genomics were identified using the following functional genomic systems, including Panther Classification System; The National Center for Biotechnology Information (https://www.ncbi.nlm.nih.gov/Structure/cdd/cddsrv.cgi?uid=KOG0710); InterPro/Pfam (integrated resource for protein families, domains and functional sites, which combine efforts of the PROSITE, PRINTS, Pfam and ProDom database projects) (https://www.ebi.ac.uk/interpro/entry/pfam/PF00011/); The Arabidopsis Information Resource (https://www.arabidopsis.org/); Genome browser: https://www.soybase.org/tools/browsers/gbrowse.html?iframe_pathname_suffix=gmax1.01, Version: Glycine max genome assembly version Glyma.Wm82.a1 (Gmax1.01).

SNP variation within these candidate genes may alter enzyme activity, gene regulation, or transport functions, thereby affecting seed sugar composition. Stable QTL regions containing pathway-related genes are particularly promising targets for marker-assisted selection and candidate gene validation. Overall, the diversity of candidate genes identified highlights the complex genetic architecture of seed sugar traits and provides a foundation for future functional studies to elucidate the mechanisms controlling sugar metabolism and accumulation.

## Conclusions

The current research identified a total of 19 significant QTL for seed sugars, among which 12 QTL are novel. Also, at least two were repeated across years, reflecting their stability and possible use for seed sugar selection by breeders. In addition to stable QTL, some QTL were colocalized between the three traits, which can be considered critical for marker-assisted selection program. Candidate genes were also identified, and they are involved in sugars metabolic pathways. Since the RIL mapping population was developed to segregate for heat tolerance, the identified SNP markers, QTL, and gene candidates provide a new resource for investigating heat tolerance. The stable QTL, and candidate genes can be used to develop KASP markers, which could help breeders to select desirable seed sugars profiling to increase soymeal value. Further research is needed to validate the relationship between QTLs and candidate genes involved in sugar metabolism and pathways. In addition, further testing in more environments is needed to enhance the stability of some QTL before they can be utilized as markers in selection.

## Data Availability

The original contributions presented in the study are publicly available. This data can be found here: https://www.ncbi.nlm.nih.gov/, SAMN60619571-SAMN60619820.

## References

[B1] Akond M. Liu S. Kantartzi S. K. Meksem K. Bellaloui N. Lightfoot D. A. . (2015). Quantitative trait loci underlying seed sugars content in “MD96-5722” by “Spencer” recombinant inbred line population of soybean. Food. Nutr. Sci. 6, 964–973. doi: 10.4236/fns.2015.611100

[B2] Bellaloui N. Reddy K. N. Bruns H. A. Gillen A. M. Mengistu A. Zobiole L. H. . (2011). “ Soybean seed composition and quality: Interactions of environment, genotype, and management practices,” in Soybeans: cultivation, uses and nutrition. Ed. Maxwell J. E. ( Nova Science Publishers, Inc, New York, USA), 1–42.

[B3] Bellaloui N. Smith J. R. Gillen A. M. Ray J. D. (2010). Effect of maturity on seed sugars as measured on near‐isogenic soybean (Glycine max) lines. Crop Sci. 50, 1978–1987. doi: 10.2135/cropsci2009.10.0596

[B4] Bellaloui N. Smith J. R. Ray J. D. Gillen A. M. (2009). Effect of maturity on seed composition in the early soybean production system as measured on near‐isogenic soybean lines. Crop Sci. 49, 608–620. doi: 10.2135/cropsci2008.04.0192

[B5] Biermann C. J. McGinnis G. D. (1989). Analysis of carbohydrates by GLC and MS (Boca Raton, FL: CRC Press).

[B6] Blackman S. A. Obendorf R. L. Leopold A. C. (1992). Maturation proteins and sugars in desiccation tolerance of developing soybean seeds. Plant Physiol. 100, 225–230. doi: 10.1104/pp.100.1.225 16652951 PMC1075542

[B7] Boydak E. Alpaslan M. Hayta M. Gerçek S. Simsek M. (2002). Seed composition of soybeans grown in the Harran region of Turkey as affected by row spacing and irrigation. J. Agric. Food. Chem. 50, 4718–4720. doi: 10.1021/jf0255331 12137503

[B8] Broman K. W. Sen S. (2009). A guide to QTL mapping with R/qtl (Vol. 46) (New York, NY: Springer).

[B9] Broman K. W. Gatti D. M. Simecek P. Furlotte N. A. Prins P. Sen Ś. . (2019). R/qtl2: software for mapping quantitative trait loci with high-dimensional data and multiparent populations. Genet. 211, 495–502. doi: 10.1534/genetics.118.301595 30591514 PMC6366910

[B10] Broman K. W. Wu H. Sen S. Churchill G. A. (2003). R/qtl: QTL mapping in experimental crosses. Bioinformatics 19 (7), 889–890. doi: 10.1093/bioinformatics/btg112 12724300

[B11] Brown A. V. Conners S. I. Huang W. Wilkey A. P. Grant D. Weeks N. T. . (2021). A new decade and new data at SoyBase, the USDA-ARS soybean genetics and genomics database. Nucleic Acids Res. 49, D1496–D1501. doi: 10.1093/nar/gkaa1107 33264401 PMC7778910

[B12] Chebrolu K. K. Fritschi F. B. Ye S. Krishnan H. B. Smith J. R. Gillman J. D. (2016). Impact of heat stress during seed development on soybean seed metabolome. Metabolomics 12, 28. doi: 10.1007/s11306-015-0941-1 41940407

[B13] Churms S. C. Zweig G. Sherma J. (1982). Handbook of chromatography (Boca Raton, FL: CRC Press).

[B14] ElSayed A. I. Rafudeen M. S. Golldack D. (2014). Physiological aspects of raffinose family oligosaccharides in plants: Protection against abiotic stress. Plant Biol. 16, 1–8. doi: 10.1111/plb.12053 23937337

[B15] Elshire R. J. Glaubitz J. C. Sun Q. Poland J. A. Kawamoto K. Buckler E. S. . (2011). A robust, simple genotyping-by-sequencing (GBS) approach for high diversity species. PloS One 6, e19379. doi: 10.1371/journal.pone.0019379 21573248 PMC3087801

[B16] Fehr W. R. Caviness C. E. (1977). Stages of soybean development. Iowa Agric. Exp. Stn. Spec. Rep. 80. (Ames: Iowa State University).

[B17] Gitzelmann R. Auriccio S. (1965). The handling of soya alpha-galactosides by a normal and a galactosemic child. Pediatr. 36, 231–235. doi: 10.1542/peds.36.2.231 14320033

[B18] González E. M. Gordon A. J. James C. L. Arrese-lgor C. (1995). The role of sucrose synthase in the response of soybean nodules to drought. J. Exp. Bot. 46, 1515–1523. doi: 10.1093/jxb/46.10.1515 12432039

[B19] Hata Y. Yamamoto M. Nakajima K. (1991). Effects of soybean oligosaccharides on human digestive organs: Estimation of fifty percent effective dose and maximum non-effective dose based on diarrhea. J. Clin. Biochem. Nutr. 10, 135–144. doi: 10.3164/jcbn.10.135 21980225 PMC3171682

[B20] Holland J. B. Nyquist W. E. Cervantes-Martínez C. T. (2003). Estimating and interpreting heritability for plant breeding: An update. Plant Breed. Rev., 9–112. doi: 10.1002/9780470650202.ch2 41939252

[B21] Horbowicz M. Obendorf R. L. (1994). Seed desiccation tolerance and storability: Dependence on flatulence-producing oligosaccharides and cyclitols—Review and survey. Seed. Sci. Res. 4, 385–405. doi: 10.1017/S0960258500002440 41822556

[B22] Hu L. Wang X. Zhang J. Florez-Palacios L. Song Q. Jiang G.-L. (2023). Genome-wide detection of quantitative trait loci and prediction of candidate genes for seed sugar composition in early mature soybean. Int. J. Mol. Sci. 24, 3167. doi: 10.3390/ijms24043167 36834578 PMC9966586

[B23] Hymowitz T. Collins F. I. (1974). Variability of sugar content in seed of Glycine max (L.) Merrill and G. soja Sieb and Zucc. Agron. J. 66, 239–240. doi: 10.2134/agronj1974.00021962006600020017x

[B24] Jamison D. R. Chen P. Hettiarachchy N. S. Miller D. M. Shakiba E. (2024). Identification of quantitative trait loci (QTL) for sucrose and protein content in soybean seed. Plants 13, 650. doi: 10.3390/plants13050650 38475496 PMC10934403

[B25] Kassem M. A. (2021). Soybean seed composition: protein, oil, fatty acids, amino acids, sugars, mineral nutrients, tocopherols, and isoflavones (Berlin/Heidelberg, Germany: Springer Nature). doi: 10.1007/978-3-030-82906-3

[B26] Keller F. Pharr D. M. (1996). “ Metabolism of carbohydrates in sinks and sources: Galactosyl-sucrose oligosaccharides,” in Photoassimilate distribution in plants and crops. Eds. Zamski E. Schaffer A. A. ( Routledge, London, UK), 157–183.

[B27] Kim H. K. Kang S. T. Cho J. H. Choung M. G. Suh D. Y. (2005). Quantitative trait loci associated with oligosaccharide and sucrose contents in soybean (Glycine max L.). J. Plant Biol. 48, 106–112. doi: 10.1007/BF03030569 41940407

[B28] Kim H. K. Kang S. T. Oh K. W. (2006). Mapping of putative quantitative trait loci controlling the total oligosaccharide and sucrose content of Glycine max seeds. J. Plant Res. 119, 533–538. doi: 10.1007/s10265-006-0004-9 16941063

[B29] Knizia D. Bellaloui N. Yuan J. Lakhssasi N. Anil E. Vuong T. . (2023). Quantitative trait loci and candidate genes that control seed sugars contents in the soybean ‘Forrest’ by ‘Williams 82’ Recombinant inbred line population. Plants 12, 3498. doi: 10.3390/plants12193498 37836238 PMC10575016

[B30] Kosambi D. D. (1943). The estimation of map distances from recombination values. Ann. Eugen. 12, 172–175. doi: 10.1111/j.1469-1809.1943.tb02321.x

[B31] Krober O. A. Cartter J. L. (1962). Quantitative interrelations of protein and nonprotein constituents of soybeans. Crop Sci. 2, 171–172. doi: 10.2135/cropsci1962.0011183X000200020028x

[B32] Kumar N. Orenday-Ortiz J. Kiszonas A. Jeffrey B. Morris C. (2019). Genetic analysis of a unique ‘Super Soft’ kernel texture phenotype in soft white spring wheat. J. Cereal Sci. 85, 162–167. doi: 10.1016/j.jcs.2018.12.003 41940325

[B33] Kumar V. Rani A. Goyal L. Pratap D. Billore S. D. Chauhan G. S. (2011). Evaluation of vegetable-type soybean for sucrose, taste-related amino acids, and isoflavone contents. Int. J. Food Prop. 14, 1142–1151. doi: 10.1080/10942911003592761 41909888

[B34] Kumar N. Smith J. R. Ray J. D. Gillman J. D. Bellaloui N. (2026). Identification of major QTLs for seed vigor and growth-related traits using a biparental population in soybean. Front. Genet. 16. doi: 10.3389/fgene.2025.1695593 41613753 PMC12849224

[B35] Liu K. (1997). Soybeans: Chemistry, technology, and utilization (NY: International Thomson Publishing, Chapman and Hall).

[B36] Liu C. Chen H. Yu Q. Gu H. Li Y. Tu B. . (2023). Identification of quantitative trait loci and candidate genes for seed sucrose and soluble sugar concentrations in soybean. Crop Sci. 63, 2976–2992. doi: 10.1002/csc2.21080 41939252

[B37] Marschner P. (2012). Marschner’s mineral nutrition of higher plants. 3rd ed. (San Diego, CA: Academic Press).

[B38] Maughan P. J. Maroof M. A. S. Buss G. R. (2000). Identification of quantitative trait loci controlling sucrose content in soybean (*Glycine max*). Mol. Breed. 6, 105–111. doi: 10.1023/A:1009628614988 41886696

[B39] Money D. Gardner K. Migicovsky Z. Schwaninger H. Zhong G. Y. Myles S. (2015). LinkImpute: fast and accurate genotype imputation for nonmodel organisms. G3 5, 2383–2390. doi: 10.1534/g3.115.021667 26377960 PMC4632058

[B40] Narayanan S. Zoong-Lwe Z. S. Gandhi N. Welti R. Fallen B. Smith J. R. . (2020). Comparative lipidomic analysis reveals heat stress responses of two soybean genotypes differing in temperature sensitivity. Plants 9, 457. doi: 10.3390/plants9040457 32260392 PMC7238245

[B41] Okada M. Ye K. (2009). Nuclear phosphoinositide signaling regulates messenger RNA export. RNA Biol. 6, 12–16. doi: 10.4161/rna.6.1.7439 19106628 PMC3704435

[B42] Paris R. L. Mengistu A. Tyler J. M. Smith J. R. (2006). Registration of soybean germplasm line DT97–4290 with moderate resistance to charcoal rot. Crop Sci. 46, 2324–2325. doi: 10.2135/cropsci2005.09.0297

[B43] Poysa M. Woodrow L. (2002). Stability of soybean seed composition and its effect on soymilk and tofu yield and quality. Food Res. Int. 35, 337–345. doi: 10.1016/S0963-9969(01)00125-9 41938126

[B44] Prasad M. Kumar N. Kulwal P. L. Röder M. Balyan H. S. Dhaliwal H. S. . (2003). QTL analysis for grain protein content using SSR markers and validation of associated markers using NILs in bread wheat. Theor. Appl. Genet. 106, 659–667. doi: 10.1007/s00122-002-1114-y 12595995

[B45] Redekar N. R. Glover N. M. Biyashev R. M. Ha B. K. Raboy V. Maroof M. A. S. (2020). Genetic interactions regulating seed phytate and oligosaccharides in soybean (Glycine max L.). PloS One 15, e0235120. doi: 10.1371/journal.pone.0235120 32584851 PMC7316244

[B46] Salari M. W. Ongom P. O. Thapa R. Nguyen H. T. Vuong T. D. Rainey K. M. (2021). Mapping QTL controlling soybean seed sucrose and oligosaccharides in a single family of soybean nested association mapping (SoyNAM) population. Plant Breed. 140, 110–122. doi: 10.1111/pbr.12883 41940437

[B47] SAS Institute (2002–2012). Statistical analysis systems (SAS) (Cary, NC, USA: SAS Institute).

[B48] Skoneczka J. A. Maroof M. A. S. Shang C. Buss G. R. (2009). Identification of candidate gene mutation associated with low stachyose phenotype in soybean line pi200508. Crop Sci. 49, 247–255. doi: 10.2135/cropsci2008.07.0403

[B49] Soybase Browser . Available online at: https://legacy.soybase.org/ (Accessed March 26, 2026).

[B50] Taira H. Tanaka H. Saito M. (1990). Effect of cultivar, seed size, and crop year on total and free sugar contents of domestic soybeans. J. Jap. Soc Food. Sci. Technol. 37, 203–213. doi: 10.3136/nskkk1962.37.3_203

[B51] TASSEL Trait Analysis by association, Evolution and Linkage. Available online at: https://www.maizegenetics.net/tassel (Accessed March 26, 2026).

[B52] Thole J. M. Nielsen E. (2008). Phosphoinositides in plants: Novel functions in membrane trafficking. Curr. Opin. Plant Biol. 11, 620–631. doi: 10.1016/j.pbi.2008.10.010 19028349

[B53] Wang S. (2012). Windows QTL Cartographer 2.5. (Raleigh, NC, USA: Department of Statistics, North Carolina State University). Available online at: http://statgen.ncsu.edu/qtlcart/WQTLCart.htm.

[B54] Wang Y. Q. Chen P. Y. Zhang B. (2014). Quantitative trait loci analysis of soluble sugar contents in soybean. Plant Breed. 133, 493–498. doi: 10.1111/pbr.12178 41940437

[B55] Wijewardana C. Reddy K. R. Bellaloui N. (2019). Soybean seed physiology, quality, and chemical composition under soil moisture stress. Food Chem. 278, 92–100. doi: 10.1016/j.foodchem.2018.11.035 30583452

[B56] Wilcox J. R. Shibles R. M. (2001). Interrelationships among seed quality attributes in soybean. Crop Sci. 41, 11–14. doi: 10.2135/cropsci2001.41111x

[B57] Xu W. Liu H. Li S. Zhang W. Wang Q. Zhang H. . (2022). Genome-wide identification of candidate genes underlying soluble sugar content in vegetable soybean (*Glycine max* L.) via association and expression analysis. J. Integr. Agric. 21 (7), 1886–1902. doi: 10.1016/j.jia.2022.08.003 PMC9387354 35991392

[B58] Xue H. Chen X. Li G. (2007). Involvement of phospholipid signaling in plant growth and hormone effects. Curr. Opin. Plant Biol. 10, 483–489. doi: 10.1016/j.pbi.2007.07.003 17709277

[B59] Zeng A. Chen P. Shi A. Wang D. Zhang B. Orazaly M. . (2014). Identification of quantitative trait loci for sucrose content in soybean seed. Crop Sci. 54, 554–564. doi: 10.2135/cropsci2013.01.0036

[B60] Zeng Z. Zhang Y. He J. Yu J. Mao X. Zheng P. . (2021). Effects of soybean raffinose on growth performance, digestibility, humoral immunity and intestinal morphology of growing pigs. Anim. Nutr. 7, 393–399. doi: 10.1016/j.aninu.2020.06.013 34258427 PMC8245804

