# Supplementary material for: Identification of genomic regions and candidate genes associated with soybean seed sugars in a RIL population
Source: Front Plant Sci. 2026 Jun 18;17:1785097. doi: 10.3389/fpls.2026.1785097 (PMC13324785; doi:10.3389/fpls.2026.1785097)
Supplement: Supplementary file 1 [file DataSheet1.pdf]

Figure S2. Below are total markers (in green and red) and total identi ied candidate genes (in black) using the following functional genomic systems, including Panther Classification System; The National Center for Biotechnology Information (https://www.ncbi.nlm.nih.gov/Structure/cdd/cddsrv.cgi?uid=KOG0710); InterPro/Pfam (integrated resource for protein families, domains and functional sites, which combine efforts of the PROSITE, PRINTS, Pfam and ProDom database projects) (https://www.ebi.ac.uk/interpro/entry/pfam/PF00011/); The Arabidopsis Information Resource (https://www.arabidopsis.org/); Genome browser: https://www.soybase.org/tools/browsers/gbrowse.html?iframe_pathname_suffix=gmax1.01, Version: Glycine max genome assembly version Glyma.Wm82.a1 (Gmax1.01).

qSu-Gm03-01- -2018 Gm03/Lg1_N 35957859_36557859

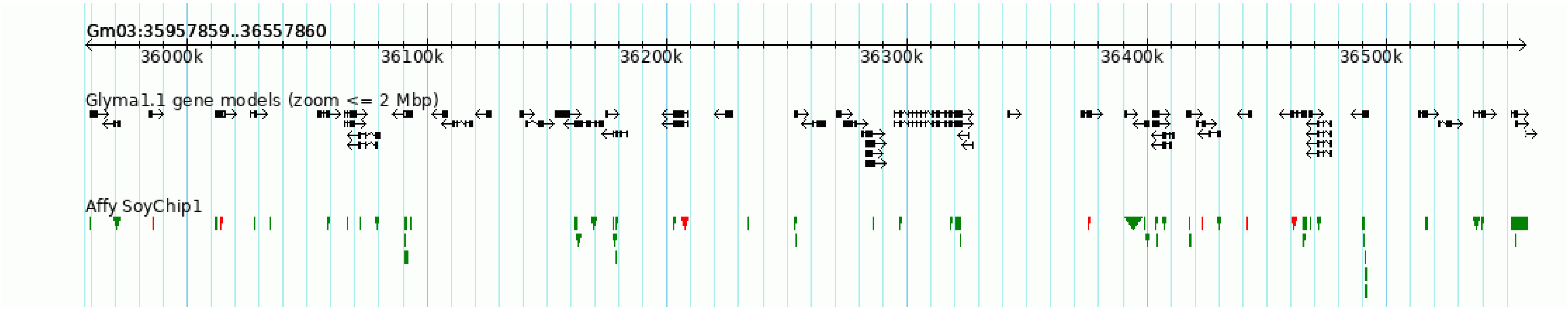

qSu-Gm05-02-2018 Gm05/Lg1_A1 41736315_42336315

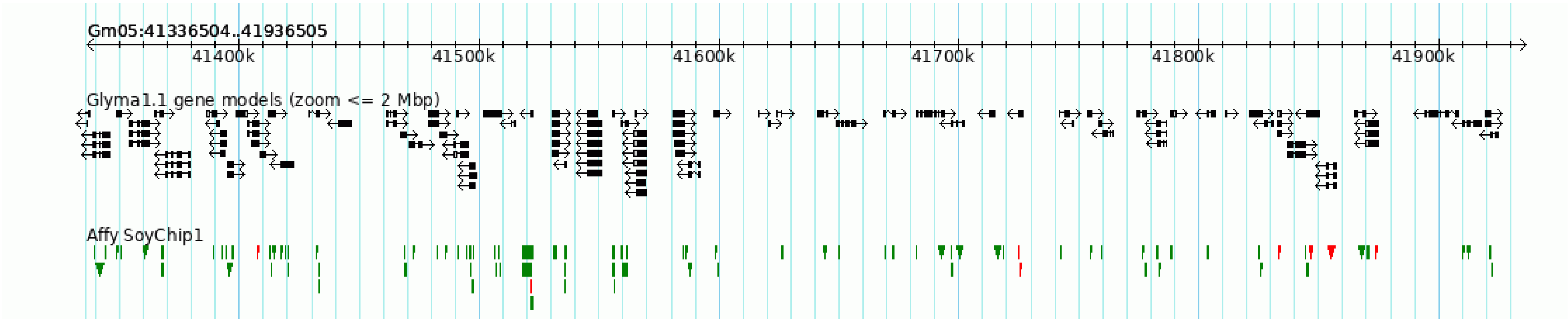

qSu-Gm11-03-2018 **Gm11/Lg1_B1 4651621_5251621**

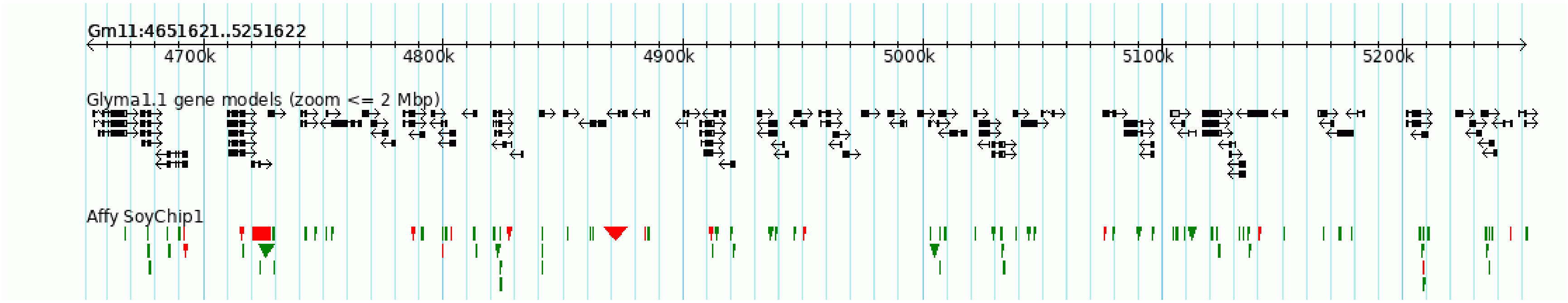

qSu-Gm19-04-2019 **Gm19Lg1 42772284_43372284**

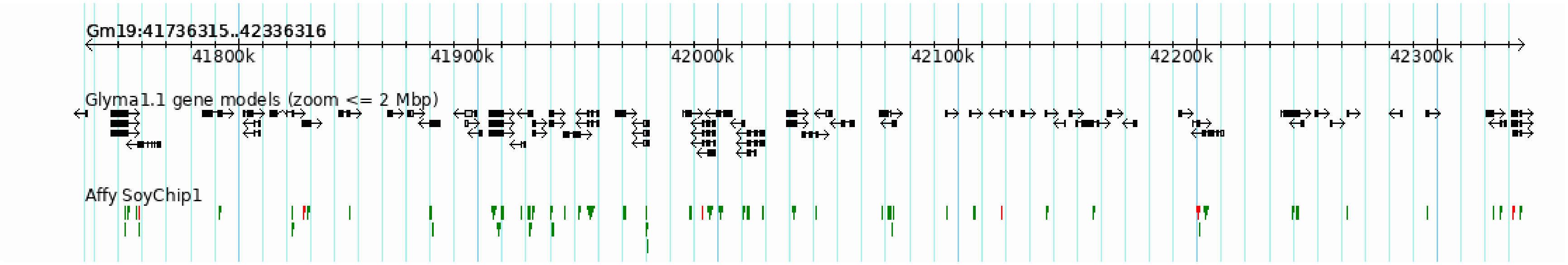

qRaf-Gm06-01-2018 Gm06L/g1_C2 19295695_19895695

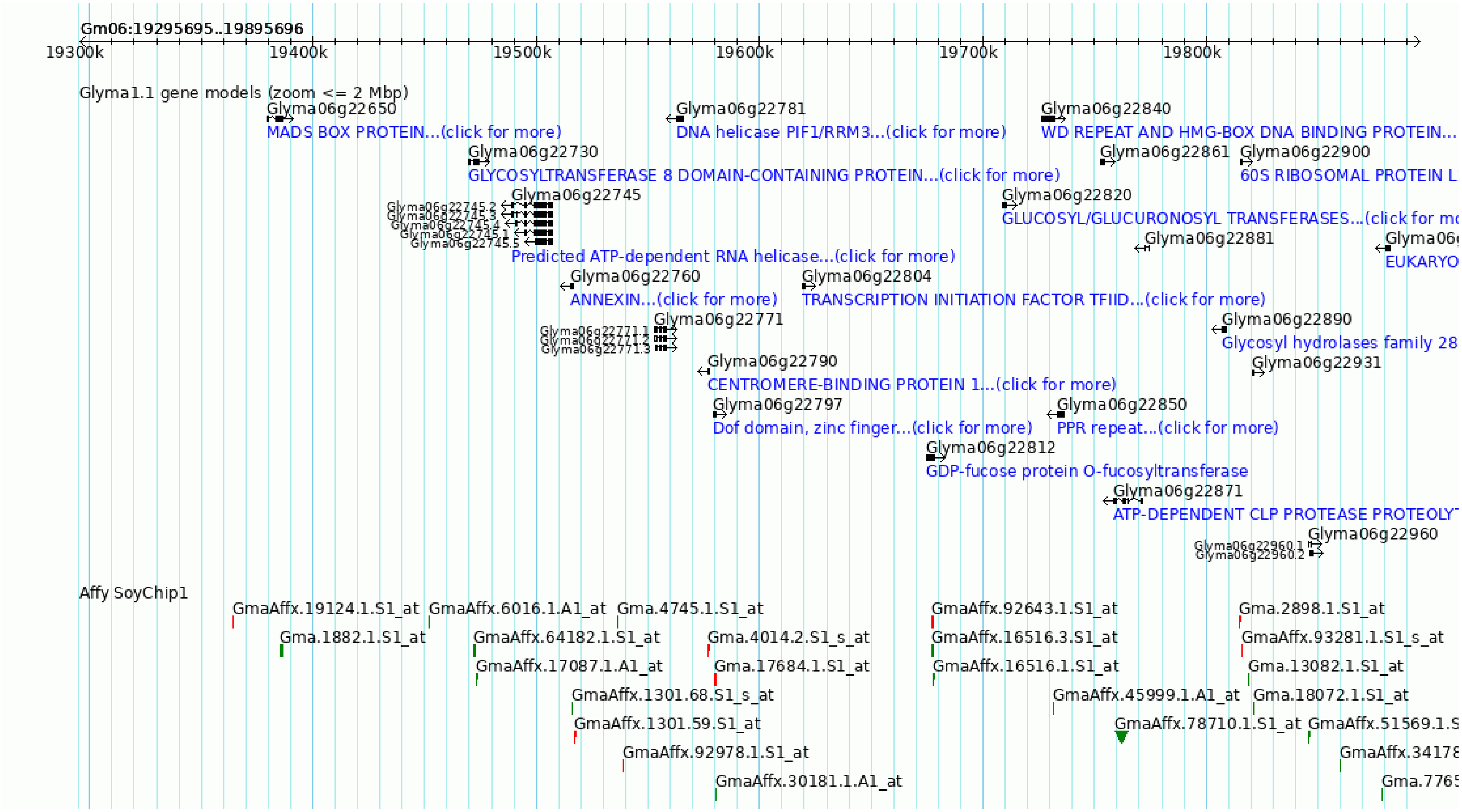
Gm06:19295695..19895696
19300k
19400k
19500k
19600k
19700k
19800k
Glyma1.1 gene models (zoom <= 2 Mbp)
Glyma06g22650
MADS BOX PROTEIN...(click for more)
Glyma06g22781
DNA helicase PIF1/RRM3...(click for more)
Glyma06g22840
WD REPEAT AND HMG-BOX DNA BINDING PROTEIN...
Glyma06g22730
GLYCOSYLTRANSFERASE 8 DOMAIN-CONTAINING PROTEIN...(click for more)
Glyma06g22861
Glyma06g22900
60S RIBOSOMAL PROTEIN L
Glyma06g22745
Predicted ATP-dependent RNA helicase...(click for more)
Glyma06g22820
GLUCOSYL/GLUCURONOSYL TRANSFERASES...(click for mo
Glyma06g22881
EUKARYO
Glyma06g22760
ANNEXIN...(click for more)
Glyma06g22804
TRANSCRIPTION INITIATION FACTOR TFIID...(click for more)
Glyma06g22771
Glyma06g22890
Glycosyl hydrolases family 28
Glyma06g22790
CENTROMERE-BINDING PROTEIN 1...(click for more)
Glyma06g22931
Glyma06g22797
Dof domain, zinc finger...(click for more)
Glyma06g22850
PPR repeat...(click for more)
Glyma06g22812
GDP-fucose protein O-fucosyltransferase
Glyma06g22871
ATP-DEPENDENT CLP PROTEASE PROTEOLY
Glyma06g22960
Affy SoyChip1
GmaAffx.19124.1.S1_at
GmaAffx.6016.1.A1_at
Gma.4745.1.S1_at
GmaAffx.92643.1.S1_at
Gma.2898.1.S1_at
Gma.1882.1.S1_at
GmaAffx.64182.1.S1_at
Gma.4014.2.S1_s_at
GmaAffx.16516.3.S1_at
GmaAffx.93281.1.S1_s_at
GmaAffx.17087.1.A1_at
Gma.17684.1.S1_at
GmaAffx.16516.1.S1_at
Gma.13082.1.S1_at
GmaAffx.1301.68.S1_s_at
GmaAffx.45999.1.A1_at
Gma.18072.1.S1_at
GmaAffx.1301.59.S1_at
GmaAffx.78710.1.S1_at
GmaAffx.92978.1.S1_at
GmaAffx.30181.1.A1_at

qRaf-Gm06-01-2019 Gm06Lg1 18181138_18781138

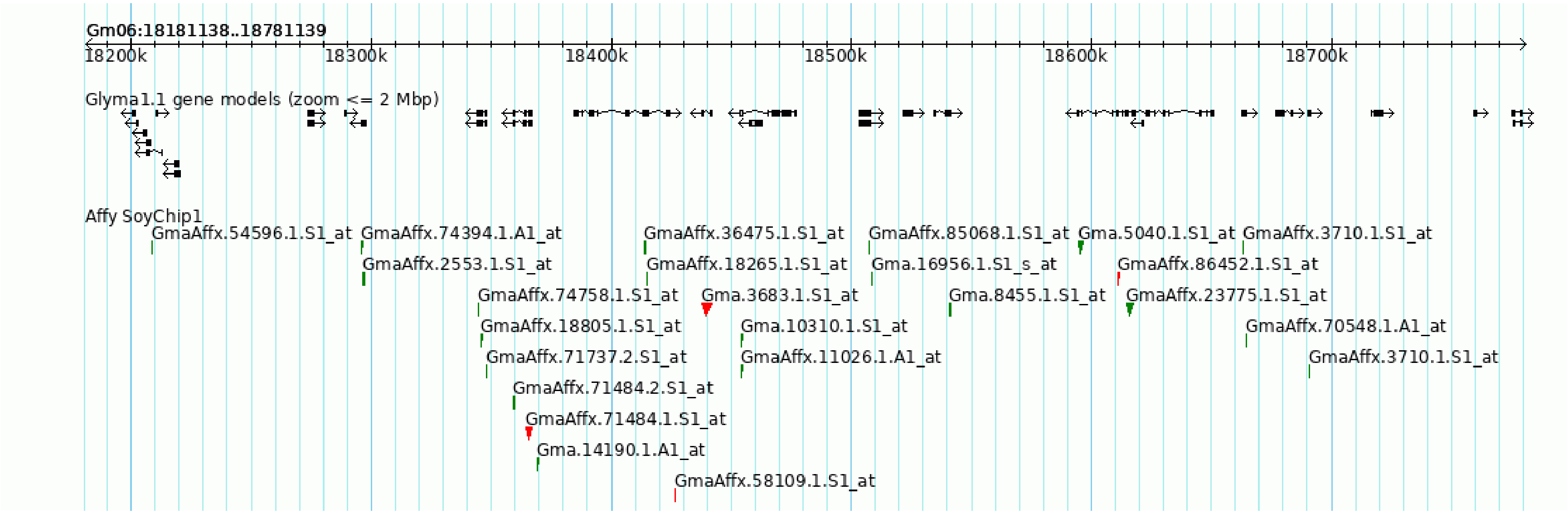

qRaf-Gm14-02-2019 Gm14Lg1E 46249574-46849574

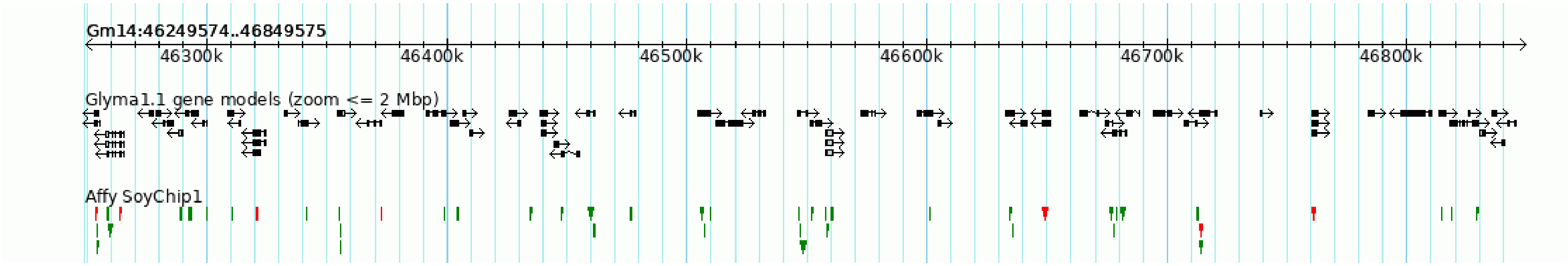

qRaf-Gm19-03-2019 Gm10Lg1 45457122_46057122

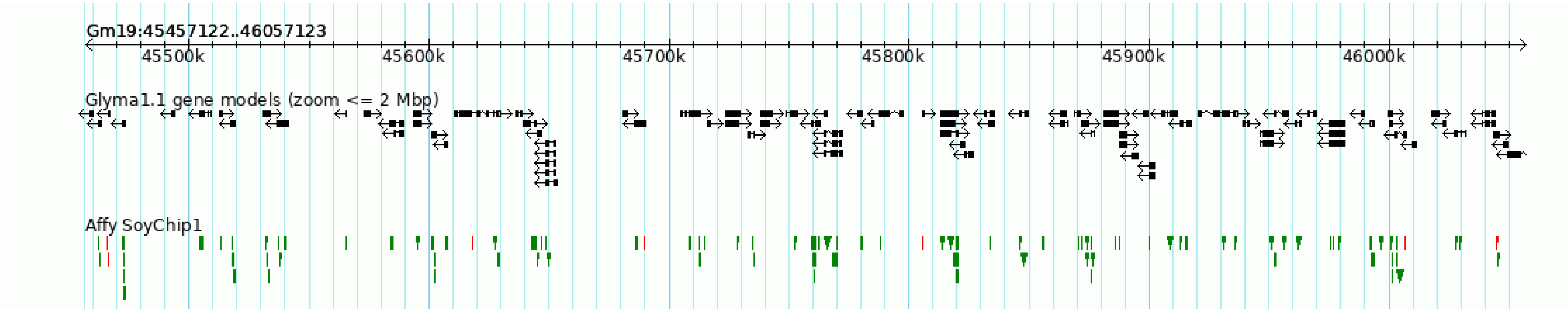

qRaf-Gm20-04-2019 Gm20Lg1 34943107-35543107

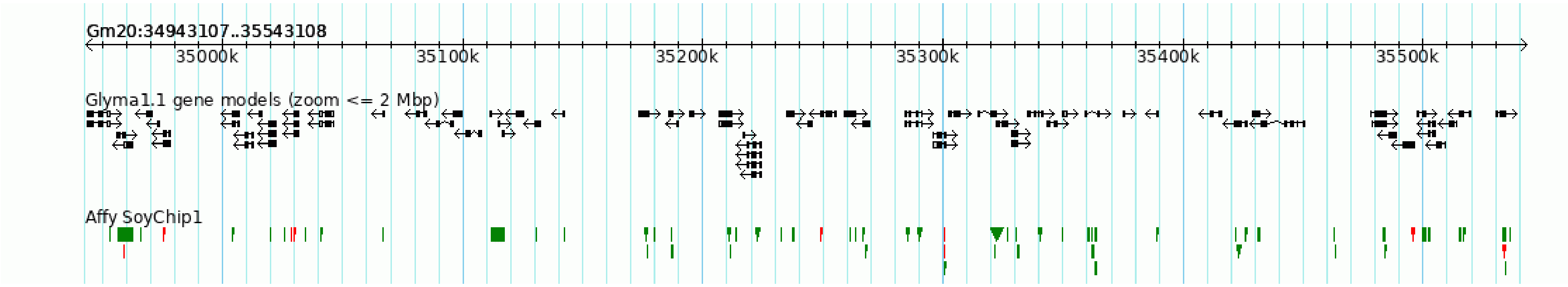

qSta-Gm06-01-2018 Gm06Lg1 43679515-44279515

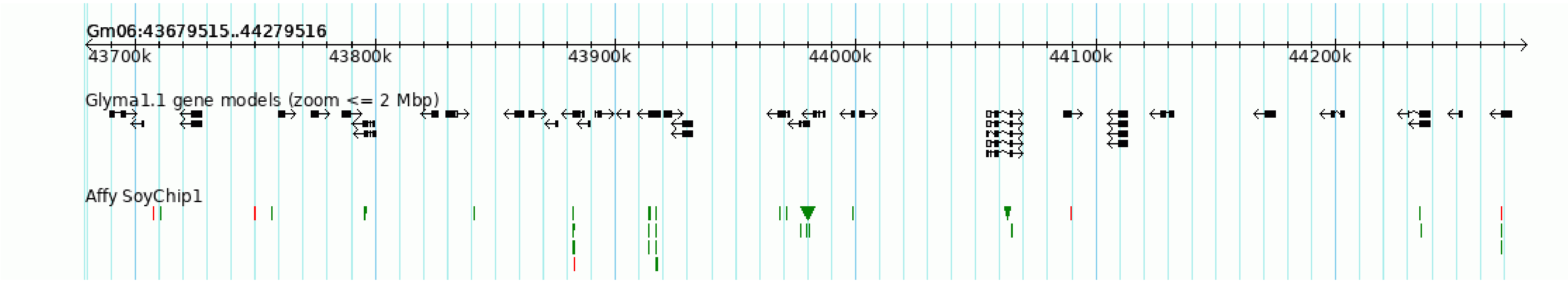

qSta-Gm19-02-2018 Gm19Lg1 42728773_43328773

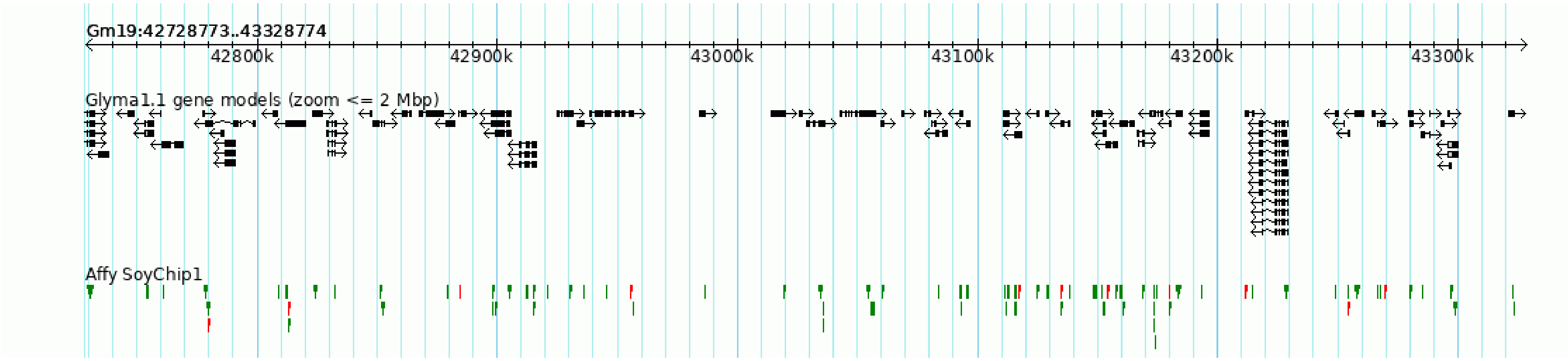

qSta-Gm03-01-2018 Gm03Lg1 35372657_35972657

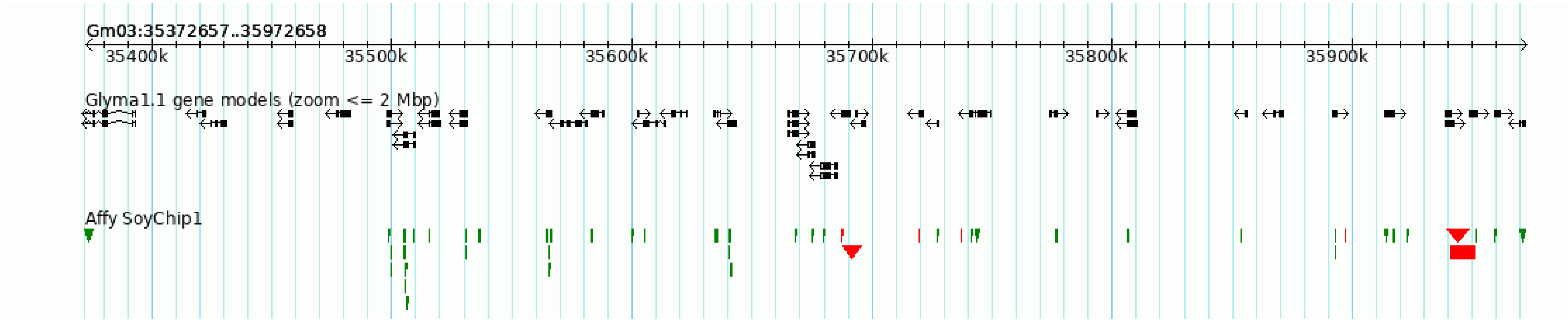

qSta-Gm04-01-2019 Gm03Lg1 35372657_35972657

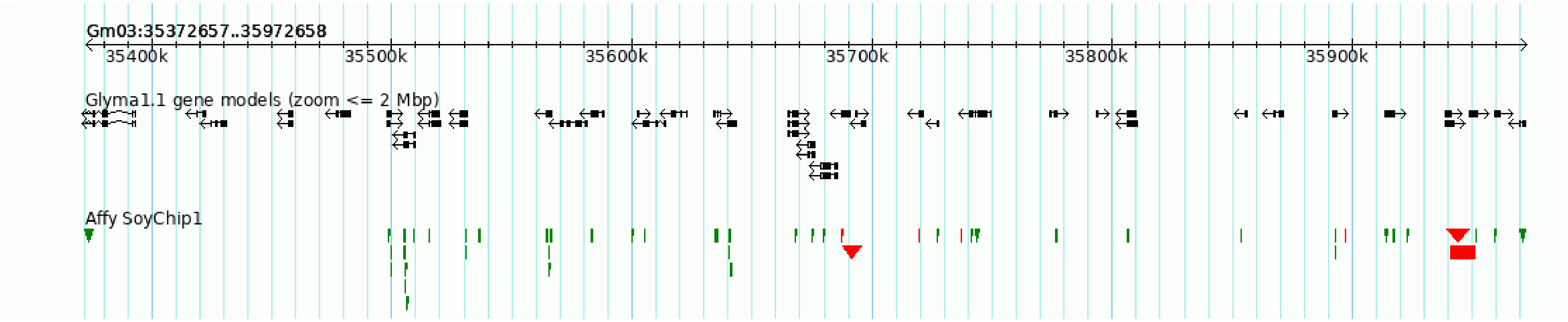

qSta-Gm13-02-2019 Gm13Lg1 27640207_28240207

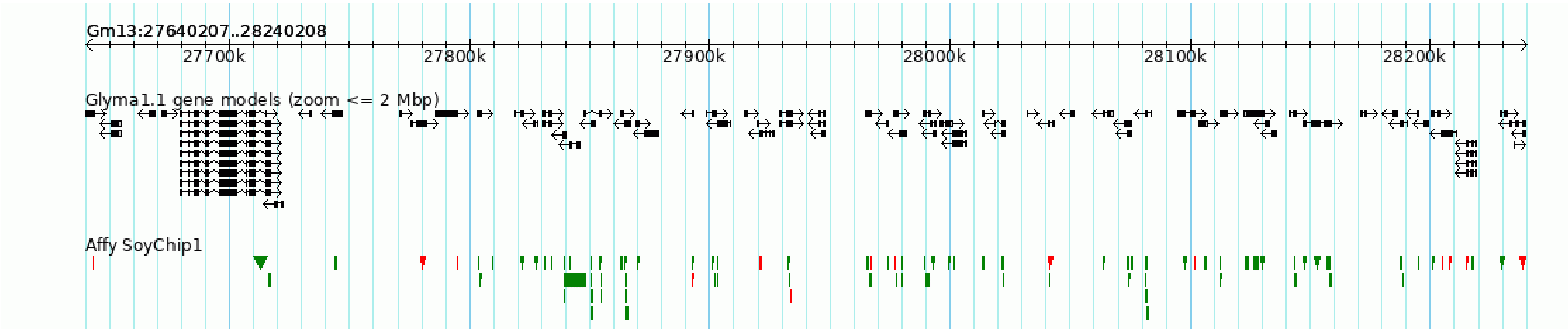

qSta-Gm19-03-2019 Gm19Lg1 46896014_47496014

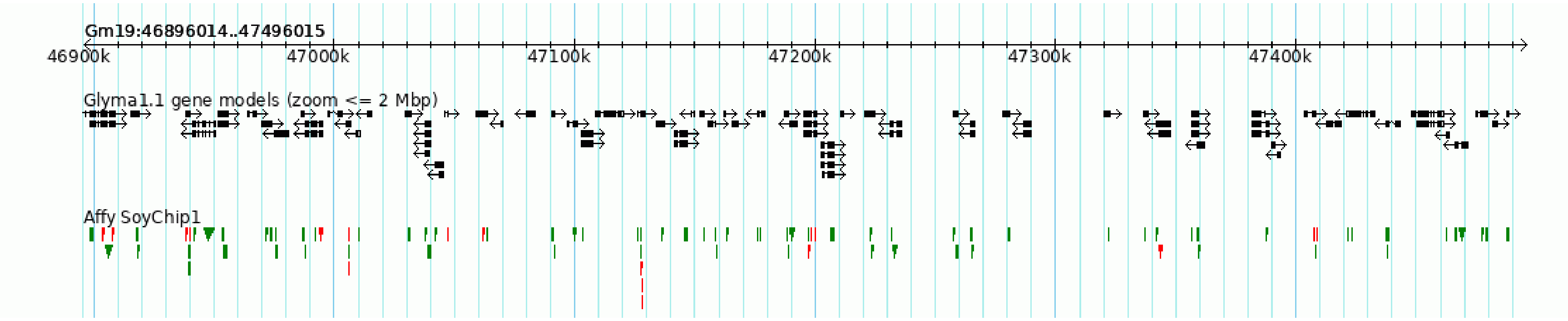

Gm19:46896014..47496015
46900k
47000k
47100k
47200k
47300k
47400k
Glyma1.1 gene models (zoom <= 2 Mbp)
Affy SoyChip1
