# Supplementary material for: Identification of genomic regions and candidate genes associated with soybean seed sugars in a RIL population
Source: Front Plant Sci. 2026 Jun 18;17:1785097. doi: 10.3389/fpls.2026.1785097 (PMC13324785; doi:10.3389/fpls.2026.1785097)
Supplement: Supplementary file 2 [file Table1.docx]

Table S1:The Means procedure in SAS to obtain means, minimum, and maximum values, and standard deviation (SD sucrose, raffinose, and stachyose for checks and parent in each year (2018 and 2019).

| **Year=2018;**  **genotype=7037-1st** |  |  |  |  | **Year=2019;**  **genotype=7037-1st** |  |  |  |  |
| --- | --- | --- | --- | --- | --- | --- | --- | --- | --- |
|  |  |  |  |  |  |  |  |  |  |
| **Variable** | **Mean** | **SD** | **Min** | **Max** | **Variable** | **Mean** | **SD** | **Min** | **Max** |
| **sucrose** | 1.45 | 0.49 | 1.10 | 1.80 | **sucrose** | 2.55 | 0.35 | 2.30 | 2.80 |
| **raffinose** | 0.82 | 0.04 | 0.79 | 0.85 | **raffinose** | 0.84 | 0.05 | 0.80 | 0.87 |
| **stachyose** | 2.75 | 0.21 | 2.60 | 2.90 | **stachyose** | 3.25 | 0.49 | 2.90 | 3.60 |
|  |  |  |  |  |  |  |  |  |  |
| **Year=2018;**  **genotype=AG4232RR** |  |  |  |  | **Year=2019; genotype=AG4232RR** |  |  |  |  |
|  |  |  |  |  |  |  |  |  |  |
| **Variable** | **Mean** | **SD** | **Min** | **Max** | **Variable** | **Mean** | **SD** | **Min** | **Max** |
| **sucrose** | 3.60 | 0.14 | 3.50 | 3.70 | **sucrose** | 3.15 | 0.64 | 2.70 | 3.60 |
| **raffinose** | 0.71 | 0.01 | 0.70 | 0.72 | **raffinose** | 0.83 | 0.06 | 0.79 | 0.87 |
| **stachyose** | 3.50 | 0.14 | 3.40 | 3.60 | **stachyose** | 3.00 | 0.57 | 2.60 | 3.40 |
|  |  |  |  |  |  |  |  |  |  |
| **Year=2018;**  **genotype=AG5606** |  |  |  |  | **Year=2019;**  **genotype=AG5606** |  |  |  |  |
|  |  |  |  |  |  |  |  |  |  |
| **Variable** | **Mean** | **SD** | **Min** | **Max** | **Variable** | **Mean** | **SD** | **Min** | **Max** |
| **sucrose** | 4.00 | 0.85 | 3.40 | 4.60 | **sucrose** | 4.05 | 0.21 | 3.90 | 4.20 |
| **raffinose** | 0.72 | 0.05 | 0.68 | 0.75 | **raffinose** | 0.80 | 0.02 | 0.78 | 0.81 |
| **stachyose** | 3.65 | 0.21 | 3.50 | 3.80 | **stachyose** | 3.40 | 0.28 | 3.20 | 3.60 |
|  |  |  |  |  |  |  |  |  |  |
| **Year=2018;**  **genotype=C4926** |  |  |  |  | **Year=2019;**  **genotype=C4926** |  |  |  |  |
|  |  |  |  |  |  |  |  |  |  |
| **Variable** | **Mean** | **SD** | **Min** | **Max** | **Variable** | **Mean** | **SD** | **Min** | **Max** |
| **sucrose** | 3.25 | 0.64 | 2.80 | 3.70 | **sucrose** | 3.20 | 0.00 | 3.20 | 3.20 |
| **raffinose** | 0.72 | 0.01 | 0.71 | 0.73 | **raffinose** | 0.79 | 0.02 | 0.77 | 0.80 |
| **stachyose** | 3.40 | 0.28 | 3.20 | 3.60 | **stachyose** | 3.60 | 0.28 | 3.40 | 3.80 |
|  |  |  |  |  |  |  |  |  |  |
| **Year=2018;**  **genotype (Parent)=**  **DS25-1** |  |  |  |  | **Year=2019;**  **genotype (Parent)=**  **DS25-1** |  |  |  |  |
|  |  |  |  |  |  |  |  |  |  |
| **Variable** | **Mean** | **SD** | **Min** | **Max** | **Variable** | **Mean** | **SD** | **Min** | **Max** |
| **sucrose** | 3.75 | 0.07 | 3.70 | 3.80 | **sucrose** | 3.30 | 0.71 | 2.80 | 3.80 |
| **raffinose** | 0.75 | 0.01 | 0.74 | 0.76 | **raffinose** | 0.87 | 0.01 | 0.86 | 0.87 |
| **stachyose** | 3.50 | 0.14 | 3.40 | 3.60 | **stachyose** | 3.20 | 0.14 | 3.10 | 3.30 |
|  |  |  |  |  |  |  |  |  |  |
| **Year=2018;**  **genotype=DS34-1** |  |  |  |  | **Year=2019;**  **genotype=DS34-1** |  |  |  |  |
|  |  |  |  |  |  |  |  |  |  |
| **Variable** | **Mean** | **SD** | **Min** | **Max** | **Variable** | **Mean** | **SD** | **Min** | **Max** |
| **sucrose** | 2.30 | 0.99 | 1.60 | 3.00 | **sucrose** | 2.90 | 0.71 | 2.40 | 3.40 |
| **raffinose** | 0.74 | 0.04 | 0.71 | 0.77 | **raffinose** | 0.85 | 0.08 | 0.79 | 0.90 |
| **stachyose** | 3.15 | 0.35 | 2.90 | 3.40 | **stachyose** | 3.15 | 0.35 | 2.90 | 3.40 |
|  |  |  |  |  |  |  |  |  |  |
| **Year=2018;**  **genotype=DS65-1** |  |  |  |  | **Year=2019;**  **genotype=DS65-1** |  |  |  |  |
|  |  |  |  |  |  |  |  |  |  |
| **Variable** | **Mean** | **SD** | **Min** | **Max** | **Variable** | **Mean** | **SD** | **Min** | **Max** |
| **sucrose** | 3.50 | 0.57 | 3.10 | 3.90 | **sucrose** | 3.60 | 0.00 | 3.60 | 3.60 |
| **raffinose** | 0.81 | 0.01 | 0.80 | 0.82 | **raffinose** | 0.87 | 0.04 | 0.84 | 0.89 |
| **stachyose** | 4.00 | 0.28 | 3.80 | 4.20 | **stachyose** | 3.45 | 0.21 | 3.30 | 3.60 |
|  |  |  |  |  |  |  |  |  |  |
| **Year=2018; genotype (Parent)=DT97-429** |  |  |  |  | **Year=2019; genotype (Parent)=DT97-429** |  |  |  |  |
|  |  |  |  |  |  |  |  |  |  |
| **Variable** | **Mean** | **SD** | **Min** | **Max** | **Variable** | **Mean** | **SD** | **Min** | **Max** |
| **sucrose** | 2.95 | 0.64 | 2.50 | 3.40 | **sucrose** | 2.65 | 0.35 | 2.40 | 2.90 |
| **raffinose** | 0.66 | 0.02 | 0.64 | 0.67 | **raffinose** | 0.79 | 0.01 | 0.78 | 0.80 |
| **stachyose** | 3.00 | 0.00 | 3.00 | 3.00 | **stachyose** | 3.05 | 0.35 | 2.80 | 3.30 |
|  |  |  |  |  |  |  |  |  |  |
| **Year=2018;**  **genotype=Dillon** |  |  |  |  | **Year=2019;**  **genotype=Dillon** |  |  |  |  |
|  |  |  |  |  |  |  |  |  |  |
| **Variable** | **Mean** | **SD** | **Min** | **Max** | **Variable** | **Mean** | **SD** | **Min** | **Max** |
| **sucrose** | 2.65 | 0.35 | 2.40 | 2.90 | **sucrose** | 3.15 | 0.49 | 2.80 | 3.50 |
| **raffinose** | 0.72 | 0.00 | 0.72 | 0.72 | **raffinose** | 0.78 | 0.01 | 0.77 | 0.78 |
| **stachyose** | 3.65 | 0.21 | 3.50 | 3.80 | **stachyose** | 3.85 | 0.35 | 3.60 | 4.10 |
|  |  |  |  |  |  |  |  |  |  |
| **Year=2018;**  **genotype=LG03-456** |  |  |  |  | **Year=2019; genotype=LG03-456** |  |  |  |  |
|  |  |  |  |  |  |  |  |  |  |
| **Variable** | **Mean** | **SD** | **Min** | **Max** | **Variable** | **Mean** | **SD** | **Min** | **Max** |
| **sucrose** | 3.95 | 0.07 | 3.90 | 4.00 | **sucrose** | 3.50 | 1.84 | 2.20 | 4.80 |
| **raffinose** | 0.73 | 0.08 | 0.67 | 0.78 | **raffinose** | 0.79 | 0.01 | 0.78 | 0.80 |
| **stachyose** | 3.40 | 0.14 | 3.30 | 3.50 | **stachyose** | 3.65 | 0.92 | 3.00 | 4.30 |
|  |  |  |  |  |  |  |  |  |  |
| **Year=2018;**  **genotype=Maverick** |  |  |  |  | **Year=2019;**  **genotype=Maverick** |  |  |  |  |
|  |  |  |  |  |  |  |  |  |  |
| **Variable** | **Mean** | **SD** | **Min** | **Max** | **Variable** | **Mean** | **SD** | **Min** | **Max** |
| **sucrose** | 3.35 | 0.35 | 3.10 | 3.60 | **sucrose** | 3.25 | 0.35 | 3.00 | 3.50 |
| **raffinose** | 0.73 | 0.11 | 0.65 | 0.80 | **raffinose** | 0.79 | 0.02 | 0.77 | 0.80 |
| **stachyose** | 3.55 | 0.64 | 3.10 | 4.00 | **stachyose** | 3.30 | 0.28 | 3.10 | 3.50 |
|  |  |  |  |  |  |  |  |  |  |
| **Year=2018;**  **genotype=Osage** |  |  |  |  | **Year=2019;**  **genotype=Osage** |  |  |  |  |
|  |  |  |  |  |  |  |  |  |  |
| **Variable** | **Mean** | **SD** | **Min** | **Max** | **Variable** | **Mean** | **SD** | **Min** | **Max** |
| **sucrose** | 2.95 | 0.07 | 2.90 | 3.00 | **sucrose** | 2.35 | 0.07 | 2.30 | 2.40 |
| **raffinose** | 0.73 | 0.13 | 0.64 | 0.82 | **raffinose** | 0.73 | 0.00 | 0.73 | 0.73 |
| **stachyose** | 3.85 | 0.21 | 3.70 | 4.00 | **stachyose** | 3.75 | 0.07 | 3.70 | 3.80 |
|  |  |  |  |  |  |  |  |  |  |
| **Year=2018;**  **genotype=Pella** |  |  |  |  | **Year=2019;**  **genotype=Pella** |  |  |  |  |
|  |  |  |  |  |  |  |  |  |  |
| **Variable** | **Mean** | **SD** | **Min** | **Max** | **Variable** | **Mean** | **SD** | **Min** | **Max** |
| **sucrose** | 86.00 | 0.00 | 86.00 | 86.00 | **sucrose** | 86.00 | 0.00 | 86.00 | 86.00 |
| **raffinose** | 2.60 | 0.14 | 2.50 | 2.70 | **raffinose** | 1.95 | 0.35 | 1.70 | 2.20 |
| **stachyose** | 0.70 | 0.06 | 0.66 | 0.74 | **stachyose** | 0.75 | 0.02 | 0.73 | 0.76 |
|  |  |  |  |  |  |  |  |  |  |
| **Year=2018;**  **genotype=Progeny** |  |  |  |  | **Year=2019;**  **genotype=Progeny** |  |  |  |  |
|  |  |  |  |  |  |  |  |  |  |
| **Variable** | **Mean** | **SD** | **Min** | **Max** | **Variable** | **Mean** | **SD** | **Min** | **Max** |
| **sucrose** | 4211.00 | 0.00 | 4211.00 | 4211.00 | **sucrose** | 4211.00 | 0.00 | 4211.00 | 4211.00 |
| **raffinose** | 3.05 | 0.07 | 3.00 | 3.10 | **raffinose** | 3.55 | 0.07 | 3.50 | 3.60 |
| **stachyose** | 0.65 | 0.04 | 0.62 | 0.67 | **stachyose** | 0.75 | 0.10 | 0.68 | 0.82 |
