# Supplementary material for: Identification of genomic regions and candidate genes associated with soybean seed sugars in a RIL population
Source: Front Plant Sci. 2026 Jun 18;17:1785097. doi: 10.3389/fpls.2026.1785097 (PMC13324785; doi:10.3389/fpls.2026.1785097)
Supplement: Supplementary file 3 [file Table2.docx]

Table S2. Candidate genes^a^ for sucrose, raffinose, and stachyose using DS25-1 × DT97-4290 RIL population.

| QTL | Name | Location | Gene annotation/gene ontology/functional genomics |
| --- | --- | --- | --- |
| qSu-01-2018 | Glyma03g28120 | Gm03:35969569..35972058 | 10 KDA HEAT SHOCK PROTEIN |
|  | Glyma03g28130 | Gm03:36011811..36015778 | MEMBRANE TRANSPORT PROTEIN |
|  | Glyma03g28260 | Gm03:36138962..36140003 | CALCIUM-BINDING PROTEIN |
|  | Glyma03g28410 | Gm03:36294501..36322871 | GLUTAMATE SYNTHASE |
| qSu-02-2018 | Glyma05g38540 | Gm05:41869789..41875270 | AUXIN RESPONSE FACTOR |
|  | Glyma05g38510 | Gm05:41844917..41850967 | ATP-DEPENDENT CLP PROTEASE |
|  | Glyma05g38560 | Gm05:41910254..41917805 | SIGNAL RECOGNITION PARTICLE 68 KDA PROTEIN |
| qSu-03-2018 | Glyma11g06640 | Gm11:4684961..4693122 | TRANSCRIPTION FACTOR MEIS1 AND RELATED HOX DOMAIN PROTEINS |
|  | Glyma11g06690 | Gm11:4727143..4729773 | CYTOCHROME P450 CYP2 SUBFAMILY |
|  | Glyma11g06750 | Gm11:4774838..4776742 | LEUCINE-RICH REPEAT RECEPTOR-LIKE PROTEIN KINASE |
|  | Glyma11g06880 | Gm11:4850212..4852030 | UDP-GLUCOSYLTRANSFERASE |
|  | Glyma11g07140 | Gm11:5006884..5009532 | TRANSCRIPTION FACTOR IIIB 90 KDA SUBUNIT |
|  | Glyma11g07340 | Gm11:5135661..5143843 | NUCLEOLAR RNA-ASSOCIATED PROTEIN |
|  | Glyma11g07310 | Gm11:5128205..5128489 | MYB-LIKE DNA-BINDING DOMAIN |
|  | Glyma11g07350 | Gm11:5150063..5151830 | TRANSCRIPTION FACTOR GATA (GATA BINDING FACTOR |
| qSu-04-2018 | Glyma19g35151 | Gm19:42707098..42714019 | NUCLEAR TRANSPORT FACTOR 2 (NTF2) FAMILY  PROTEIN / RNA RECOGNITION MOTIF (RRM)-CONTAINING PROTEIN) |
|  | Glyma19g35236 | Gm19:42778315..42799255 | ATP-BINDING CASSETTE TRANSPORTER |
|  | Glyma19g35401 | Gm19:42933164..42936374 | SUGAR KINASE |
|  | Glyma19g35480 | Gm19:43068593..43069234 | RESPONSE REGULATOR RECEIVER DOMAIN) |
|  | Glyma19g35670 | Gm19:43166744..43169591 | RNA RECOGNITION MOTIF. (A.K.A. RRM, RBD, OR RNP DOMAIN |
| qRaf-01-2018 | Glyma06g22730 | Gm06:19469580..19474113 | GLYCOSYLTRANSFERASE 8 DOMAIN-CONTAINING PROTEIN |
|  | Glyma06g22820 | Gm06:19708865..19710648 | GLUCOSYL/GLUCURONOSYL TRANSFERASES |
|  | Glyma06g22812 | Gm06:19674533..19678514 | GDP-FUCOSE PROTEIN O-FUCOSYLTRANSFERASE |
|  | Glyma06g22812 | Gm06:19674533..19678514 | GDP-FUCOSE PROTEIN O-FUCOSYLTRANSFERASE |
| qRaf-01-2018 | Glyma20g25170 | Gm20:34912898..34913895 | ZINC FINGER PROTEIN WITH KRAB AND SCAN DOMAINS |
|  | Glyma20g25210 | Gm20:34956278..34959486 | CORE-2/I-BRANCHING ENZYME |
|  | Glyma20g25191 | Gm20:34929356..34930720 | RNA RECOGNITION MOTIF. (A.K.A. RRM, RBD, OR RNP DOMAIN |
|  | Glyma20g25200 | Gm20:34943755..34953280 | METALLO-BETA-LACTAMASE RELATED |
|  | Glyma20g25291 | Gm20:35009152..35013448 | LEUCINE-RICH REPEAT RECEPTOR-LIKE PROTEIN KINASE |
|  | Glyma20g25580 | Gm20:35243672..35245204 | AUX/IAA FAMILY |
|  | Glyma20g25712 | Gm20:35343272..35347269 | CCCH ZINC FINGER/TIS11-RELATED |
|  | Glyma20g25790 | Gm20:35429047..35431858 | FRUCTOSE-BISPHOSPHATE ALDOLASE |
|  | Glyma20g25800 | Gm20:35432475..35451008 | SUBFAMILY NOT NAMED); ATP-DEPENDENT RNA HELICASE A |
| qRaf-01-2019 | Glyma06g21730 | Gm06:18295701..18298404 | DESCRIPTION UNAVAILABLE)); C2H2-LIKE ZINC FINGER PROTEIN |
|  | Glyma06g21883 | Gm06:18457856..18462719 | RNA RECOGNITION MOTIF. (A.K.A. RRM, RBD, OR RNP DOMAIN) |
|  | Glyma06g21910 | Gm06:18521860..18525876 | ATP-DEPENDENT CLP PROTEASE |
|  | Glyma06g22030 | Gm06:18759337..18760652 | GLYCOSYL HYDROLASES FAMILY 28 |
| qRaf-02-2019 | Glyma14g37170 | Gm14:46444640..46446280 | UDP-GLUCOSYLTRANSFERASE |
|  | Glyma14g37180 | Gm14:46447731..46455140 | RNA-BINDING PROTEIN |
|  | Glyma14g37241 | Gm14:46511849..46523095 | ATP-BINDING CASSETTE TRANSPORTER |
|  | Glyma14g37260 | Gm14:46546240..46549920 | SUGAR KINASE |
|  | Glyma14g37522 | Gm14:46817720..46829863 | FRUCTOSE-2,6-BISPHOSPHATASE |
|  | Glyma14g37560 | Gm14:46842224..46845706 | ZINC/IRON TRANSPORTER, PLANT AND YEAST |
|  | Glyma14g37540 | Gm14:46835726..46836988 | NITRATE, FROMATE, IRON DEHYDROGENASE |
| qRaf-03-2019 | Glyma19g38854 | Gm19:45715838..45717982 | FOLATE SYNTHESIS PROTEINS |
|  | Glyma19g38890 | Gm19:45738315..45742815 | SERINE/THREONINE-PROTEIN KINASE |
|  | Glyma19g39320 | Gm19:46043630..46045813 | FAMILY NOT NAMED |
| qRaf-04-2019 | Glyma20g25580 | Gm20:35243672..35245204 | AUX/IAA FAMILY |
|  | Glyma20g25640 | Gm20:35284346..35291382 | LIPID PHOSPHATE PHOSPHATASE |
|  | Glyma20g25580 | Gm20:35243672..35245204 | AUX/IAA FAMILY |
|  | Glyma20g25530 | Gm20:35194535..35196239 | RETICULON-RELATED (PLANT |
|  | Glyma20g25660 | Gm20:35302560..35307176 | AMINO ACID PERMEASE-RELATED |
| qSta-01-2018 | Glyma06g40601 | Gm06:43773270..43776039 | LEUCINE-RICH REPEAT RECEPTOR-LIKE PROTEIN KINASE |
|  | Glyma06g40610 | Gm06:43785937..43789798 | LEUCINE-RICH REPEAT RECEPTOR-LIKE PROTEIN KINASE |
|  | Glyma06g40730 | Gm06:43891776..43894380 | 1,4-BENZOQUINONE REDUCTASE-LIKE, TRP REPRESSOR  BINDING PROTEIN-LIKE/PROTOPLAST-SECRETED PROTEIN) |
|  | Glyma06g40800 | Gm06:43982736..43986933 | ALDO/KETO REDUCTASE) |
|  | Glyma06g40810 | Gm06:43998473..43999283 | 1,4-BENZOQUINONE REDUCTASE-LIKE,  TRP REPRESSOR BINDING PROTEIN-LIKE/PROTOPLAST-SECRETED PROTEIN |
|  | Glyma06g40860 | Gm06:44086613..44089781 | GH3 AUXIN-RESPONSIVE PROMOTER |
|  | Glyma06g40880 | Gm06:44109515..44112977 | LEUCINE-RICH REPEAT RECEPTOR-LIKE PROTEIN KINASE |
| qSta-02-2018 | Glyma19g35251 | Gm19:42786701..42790760 | ATP-BINDING CASSETTE TRANSPORTER (PDR) |
|  | Glyma19g35295 | Gm19:42847114..42848029 | RESPONSE REGULATOR OF TWO-COMPONENT SYSTEM |
|  | Glyma19g35270 | Gm19:42811986..42819964 | ATP-BINDING CASSETTE TRANSPORTER (PDR) |
|  | Glyma19g35340 | Gm19:42878781..42882076 | ALCOHOL DEHYDROGENASE, CLASS III |
|  | Glyma19g35401 | Gm19:42933164..42936374 | SUGAR KINASE |
|  | Glyma19g35430 | Gm19:43014178..43020034 | PHOSPHATIDYLINOSITOL N-ACETYLGLUCOSAMINYLTRANSFERASE  SUBUNIT P (DOWN SYNDROME CRITICAL REGION PROTEIN 5)-RELATED) |
|  | Glyma19g35480 | Gm19:43068593..43069234 | RESPONSE REGULATOR RECEIVER DOMAIN |
|  | Glyma19g35560 | Gm19:43115295..43118308 | HEAT SHOCK PROTEIN 70KDA |
|  | Glyma19g35670 | Gm19:43166744..43169591 | SUBFAMILY NOT NAMED); PF00076 (RNA RECOGNITION MOTIF.  (A.K.A. RRM, RBD, OR RNP DOMAIN)) |
| qSta-01-2019 | Glyma03g27770 | Gm03:35564260..35566476 | CYTOCHROME P450 CYP4/CYP19/CYP26 SUBFAMILIES |
|  | Glyma03g27790 | Gm03:35570186..35581178 | UBIQUITIN C-TERMINAL HYDROLASE |
|  | Glyma03g27800 | Gm03:35582912..35588047 | H+/OLIGOPEPTIDE SYMPORTER;) |
|  | Glyma03g27830 | Gm03:35604981..35613864 | OLIGOPEPTIDE TRANSPORTER-RELATED |
|  | Glyma03g27840 | Gm03:35616234..35623127 | H+/OLIGOPEPTIDE SYMPORTER |
|  | Glyma03g27865 | Gm03:35640069..35643599 | TRANSCRIPTION FACTOR |
| qSta-02-2019 | Glyma13g24260 | Gm13:27635616..27643692 | GUANYLATE BINDING PROTEIN |
|  | Glyma13g24270 | Gm13:27650581..27655157 | X-BOX TRANSCRIPTION FACTOR-RELATED |
|  | Glyma13g24305 | Gm13:27679361..27716855 | VACUOLAR PROTEIN SORTING-ASSOCIATED PROTEIN (VPS13;) |
|  | Glyma13g24360 | Gm13:27775790..27781750 | SOLUTE CARRIER FAMILY 35 |
|  | Glyma13g24340 | Gm13:27742731..27747108 | LEUCINE-RICH REPEAT RECEPTOR-LIKE PROTEIN KINASE;) |
|  | Glyma13g24440 | Gm13:27839025..27840078 | HEAT-SHOCK PROTEIN 17); MOLECULAR CHAPERONE;  SMALL HEAT-SHOCK PROTEIN HSP26/HSP42 |
|  | Glyma13g24440 | Gm13:27839025..27840078 | HEAT-SHOCK PROTEIN 17 |
|  | Glyma13g24461 | Gm13:27847776..27854806 | HEAT-SHOCK PROTEIN 17 |
|  | Glyma13g24420 | Gm13:27830405..27834313 | DROUGHT INDUCED 19 PROTEIN (DI19), ZINC-BINDING |
|  | Glyma13g24420 | Gm13:27830405..27834313 | PF05605 (DROUGHT INDUCED 19 PROTEIN (DI19), ZINC-BINDING;  AT3G05700.1 (DROUGHT-RESPONSIVE FAMILY PROTEIN |
|  | Glyma13g24470 | Gm13:27850355..27853113 | UBIQUITIN); KOG0005 (UBIQUITIN-LIKE PROTEIN |
|  | Glyma13g24580 | Gm13:27929285..27934547 | MITOCHONDRIAL CARNITINE-ACYLCARNITINE CARRIER PROTEIN |
|  | Glyma13g24860 | Gm13:28171233..28173685 | HEAT SHOCK TRANSCRIPTION FACTOR |
| Gm19Lg1_L | Glyma19g40520 | Gm19:46894669..46908907 | SUBFAMILY NOT NAMED);METALLOPROTEINASE-RELATED COLLAGENASE PM5 |
|  | Glyma19g40550 | Gm19:46915407..46918937 | RAFFINOSE SYNTHASE OR SEED IMBIBITION PROTEIN SIP1 |
|  | Glyma19g40560 | Gm19:46938033..46940019 | WRKY DNA -BINDING DOMAIN |
|  | Glyma19g40620 | Gm19:46986374..46987701 | IRON-SULFUR CLUSTER SCAFFOLD PROTEIN NFU-RELATED |
|  | Glyma19g40640 | Gm19:46997217..47003370 | OXIDOREDUCTASE, 2OG-FE(II) OXYGENASE FAMILY PROTEIN |
|  | Glyma19g40680 | Gm19:47029812..47032065 | GALACTINOL SYNTHASE-RELATED |
|  | Glyma19g40720 | Gm19:47059021..47063814 | ANION EXCHANGE PROTEIN-RELATED |
|  | Glyma19g40740 | Gm19:47080241..47083418 | GLYCOSYL HYDROLASES FAMILY 28 |
|  | Glyma19g40920 | Gm19:47195471..47200849 | PROTEIN TRANSPORT PROTEIN SEC23 |
|  | Glyma19g40970 | Gm19:47264459..47266048 | AUX/IAA FAMILY |
|  | Glyma19g40820 | Gm19:47133638..47137417 | SERINE/THREONINE  PROTEIN KINASE |
|  |  |  |  |

^a^The above gene annotation/functional genomics were identified using the following functional genomic systems, including Panther Classification System; The National Center for Biotechnology Information (<https://www.ncbi.nlm.nih.gov/Structure/cdd/cddsrv.cgi?uid=KOG0710>); InterPro/Pfam (integrated resource for protein families, domains and functional sites, which combine efforts of the PROSITE, PRINTS, Pfam and ProDom database projects) (<https://www.ebi.ac.uk/interpro/entry/pfam/PF00011/>); The Arabidopsis Information Resource (<https://www.arabidopsis.org/>); Genome browser: <https://www.soybase.org/tools/browsers/gbrowse.html?iframe_pathname_suffix=gmax1.01>, Version: Glycine max genome assembly version Glyma.Wm82.a1 (Gmax1.01).
